# FAIRness at all costs? Practical limitations in applying the FAIR principles to plant genetic resources data

**DOI:** 10.3389/fpls.2026.1888670

**Published:** 2026-08-06

**Authors:** Catherine Hazel M. Aguilar, Markus Oppermann, Stephan Weise

**Affiliations:** Genebank Department, Leibniz Institute of Plant Genetics and Crop Plant Research (IPK), Seeland, Germany

**Keywords:** data governance, EURISCO, FAIR data, federated information systems, genebank, plant genetic resources

## Abstract

Modern scientific infrastructure increasingly depends on standardized data governance frameworks, with FAIR (Findable, Accessible, Interoperable, Reusable) principles now widely adopted across research domains. This study investigates how FAIR principles are implemented in plant genetic resources (PGR) information systems by assessing the European Search Catalogue for Plant Genetic Resources (EURISCO) against the Research Data Alliance (RDA) FAIR Data Maturity Model indicators. As a large, federated catalogue containing data on 2.1 million accessions from 43 countries, EURISCO provides a strong empirical basis for evaluating FAIR implementation in distributed biological data systems. The assessment reveals uneven compliance across FAIR dimensions. Human-oriented findability and manual accessibility are comparatively strong, supported by a consolidated catalogue interface, open web access, and standardized passport data using Multi-Crop Passport Descriptors (MCPD v2.1). Persistent identification remains partial because the Digital Object Identifiers (DOI) currently cover only a subset of accessions and primarily identify physical germplasm instead of versioned EURISCO data record. Machine-actionability and semantic interoperability exhibit the most pronounced gaps, reflecting limited use of machine-readable formats, formal vocabularies and explicit links to external knowledge resources. The assessment also highlights domain-specific constraints, including tight coupling of physical collections and digital records, nationally distributed governance, heterogeneous institutional capacities, and long-standing legacy data integration requirements. Evaluation across *ex situ* passport, *in situ* crop wild relative, and phenotypic data domains indicates implementation gradients aligned with standardization maturity and documentation complexity. Standardized FAIR metrics only partially capture these constraints inherent to PGR information systems. Effective data governance must therefore balance FAIR ambitions with practical limits, rather than assume that generic frameworks can be applied uniformly across all contexts. Domain-calibrated approaches that prioritize scientific utility and fitness-for-purpose over exhaustive metric-based compliance may ultimately better support complex biological data systems underpinning global agricultural research.

## 1 Introduction

The concept of scientific data as knowledge commons has significantly altered approaches to data sharing and collaboration among institutions, leading to increased scientific innovations and, consequently, better solutions to intractable global challenges (Hess and Ostrom, 2005; Farley et al., 2024). This commons-based perspective is gaining traction as researchers increasingly acknowledge the value of systematically structured, publicly accessible datasets that allow for independent verification of findings, exploration of novel analytical approaches, and cumulative knowledge-building, all while circumventing redundant, resource-intensive data collection (Mandeville et al., 2021; Ugochukwu and Phillips, 2024). Drawing on this rationale, research institutions, government agencies and multilateral organizations are placing growing emphasis on scientific data as a public good, institutionalizing open-data practices and supporting frameworks that strengthen data stewardship throughout the research lifecycle (Taylor, 2016; Osimo and Pizzamigliio, 2023; Kumar et al., 2024).

One prominent framework is the FAIR Guiding Principles, which articulate four key data attributes, viz., Findable, Accessible, Interoperable, Reusable (Wilkinson et al., 2016). A distinguishing feature of FAIR is its applicability to both researcher-led methods and automated computational workflows, with an emphasis on optimal machine-actionability. As concurrent technological innovations yield unprecedented volumes of digital information relevant to both theoretical research and pressing societal challenges, research communities have been developing discipline-specific standards (Yilmaz et al., 2011; Papoutsoglou et al., 2020; Pessanha Santos, 2023), ontologies (Arnaud et al., 2020) and dedicated data infrastructures (Arita et al., 2021; Karsch-Mizrachi et al., 2025) that reflect FAIR’s overarching goals. Although originally intended to guide broad-scale digital data stewardship, FAIR soon became a widely accepted benchmark for assessing and refining data management procedures. Its wide adoption is evidenced by the mandates of major funding entities (e.g., the European Commission) that increasingly require FAIR-compliant data management protocols (European Commission, 2017) as a means to maximize research investment impact, as well as in large-scale initiatives such as the European Open Science Cloud (EOSC), which further integrate FAIR into open science ecosystems (European Commission Expert Group on FAIR Data, 2018).

However, the practical implementation of FAIR can prove to be complex, and requires substantial investments in data curation, (meta)data standardization, semantic frameworks and the alignment of diverse protocols (Jacobsen et al., 2020; Ugochukwu and Phillips, 2024). It is increasingly apparent that achieving a truly FAIR data environment requires concerted efforts not just at the dataset level, but across the diverse network of data aggregators, repositories, and services (Koers et al., 2020). These considerations become particularly salient in multifaceted domains such as plant genetic resources (PGR) conservation, management, and utilization, where digital data are tightly coupled with physical biological materials. Dynamic, living PGR collections are subject to periodic regeneration, distribution, exchange and management across multiple genebanks over time, with immediate operational consequences when data become decoupled from the material. PGR systems also involve varied legal frameworks across multiple jurisdictions, and evolving infrastructures that facilitate cross-border exchange of both materials and related information.

PGR collections, known repositories of genetic diversity, are indispensable for ensuring global food security and agro-ecological resilience, especially under growing pressures from climate variability, land-use changes, emerging pathogens, biodiversity loss and increasing population demands (Halewood et al., 2018; Volk et al., 2021; Pathirana and Carimi, 2022). Despite burgeoning needs worldwide, PGR utilization remains sub-optimal due to mounting challenges in data acquisition, handling, management and sharing across institutions and disciplines, among other limiting factors (Anglin et al., 2018).

Unlike many scientific domains where FAIR implementation builds upon recently developed digital infrastructures, PGR information systems trace their roots to longstanding institutional practices centered primarily on maintaining viable physical genetic material. These systems have evolved organically over decades in a decentralized, incremental manner (Anglin et al., 2018; van Hintum et al., 2021), responding to localized collection management imperatives in the absence of coordinated, inter-institutional strategy. This has shaped a PGR data landscape characterized by heterogeneity in descriptors, vocabularies, levels of granularity across data types, and organizational frameworks, compounded by uneven data quality, incomplete or fragmented metadata, and disparate technological platforms that together sustain persistent data siloes and constrain discovery and analysis (Halewood et al., 2018; Anglin et al., 2018; Volk et al., 2021).

The European Search Catalogue for Plant Genetic Resources (EURISCO) (Weise et al., 2017; Kreide et al., 2019; Kotni et al., 2023) (https://www.ecpgr.org/eurisco/) represents one of the most extensive catalogues for PGR globally, consolidating data from across Europe and adjacent countries to improve discoverability and facilitate access to, exchange and utilization of diverse germplasm. Established in 2003 under the aegis of the European Cooperative Programme for Plant Genetic Resources (ECPGR) and currently hosted by the Leibniz Institute of Plant Genetics and Crop Plant Research (IPK), EURISCO aggregates accession-level information on more than two million accessions from over 400 institutions across 43 countries. Initially focused on *ex situ* passport and phenotypic data, the catalogue was extended in 2024 to include *in situ* crop wild relatives (CWR) population data, bringing together accession−level records and population−level observations generated under diverse institutional arrangements. As a central entry point built on nationally curated inventories, EURISCO integrates these datasets through a single discovery interface while remaining dependent on the documentation practices, database infrastructures and governance frameworks of its data providers (Weise et al., 2020). EURISCO therefore represents a well-defined case for examining how FAIR principles are applied at infrastructure level in a globally significant domain shaped by diverse institutional practices and frameworks.

This paper assesses EURISCO’s compliance with the FAIR principles, examining how biological, structural and legal factors influence its level of compliance to established rubrics. It evaluates potential gaps and tensions that emerge while EURISCO balances centralized data integration with diverse governance structures, and considers whether domain-specific implementations of FAIR principles may be warranted for complex, multifaceted systems.

## 2 Materials and methods

The Research Data Alliance (RDA) FAIR Data Maturity Model, with its 41 indicators across FAIR dimensions (Bahim et al., 2020), was utilized for this assessment due to its comprehensive scope and emphasis on both technical implementation and knowledge representation aspects. While originally designed for dataset-level evaluation, the model explicitly acknowledges applicability to “data-related algorithms, tools, workflows, protocols and other data-related services” managed as digital objects, which provides a methodological basis for extending its use to infrastructures that operate as integrated information systems. This extended scope has already been tested at infrastructure level, for example in the evaluation of the Virtual Atomic and Molecular Data Centre (VAMDC) e-infrastructure, which reported that this framework originally aimed at standalone datasets or repositories also worked effectively for a distributed and complex e-infrastructure (Zwölf and Moreau, 2023). This precedent supports its use here for assessing EURISCO as an integrated information system while accommodating the distinct characteristics of the multiple data domains it manages.

In order to apply this framework to EURISCO, a structured adaptation process was carried out, involving three main steps: (1) contextual interpretation of each indicator within the PGR domain, drawing on established domain knowledge and practices; (2) development of EURISCO-specific evaluation criteria for each applicable indicator, considering both the infrastructure’s design parameters and domain-specific requirements; and (3) establishment of evidence requirements for each indicator, defining what would constitute compliance at different levels. The assessment considered the three distinct data domains managed within EURISCO, i.e., *ex situ* passport, *in situ* CWR passport, and phenotypic data. *Ex situ* passport data describe individual genebank accessions conserved in collections (e.g., accession identifiers, taxonomy, origin, storage status). *In situ* CWR passport data instead refer to occurrences or populations of CWR in natural or semi-natural habitats, emphasizing georeferenced locations, population descriptors, and conservation or management context. Phenotypic data comprise trait observations generated in characterization and evaluation experiments, typically organized as plot- or plant-level measurements linked to accessions, environments, and experimental protocols. These three data domains differ in granularity, degree of standardization, and update dynamics. The RDA model’s flexibility for community-specific adaptation accommodates these variations while maintaining consistent evaluation criteria across the infrastructure, as illustrated by its application to the BEXIS2 biodiversity data management system, where a self-assessment using the model showed strong conformance with FAIR indicators across heterogeneous ecological and environmental datasets (Chamanara et al., 2021).

The assessment methodology employed a detailed examination protocol mapping each indicator to specific EURISCO features and functions. This guided the systematic review of EURISCO’s public interface, functionality, access mechanisms, (meta)data structures and documentation, with direct system testing conducted for applicable indicators. Because EURISCO is continuously updated, the assessment was treated as a time-bound evaluation. The system review and independent scoring were conducted between December, 2024 and February, 2025, and consensus review was completed by March 2025. Quantitative evaluation utilized a standardized 1–5 scale for each indicator. A score of 1 indicated no compliance, while a score of 5 represented exemplary implementation. This scale was applied with clearly defined criteria for each level to ensure consistency across the assessment. For each applicable indicator and data domain, three evaluators independently assigned an initial score and recorded a brief rationale. This captured both the evidence considered and the evaluator’s interpretation of the RDA indicator in the EURISCO/PGR context. Final scores were assigned through iterative consensus review. For all non-identical initial scores, the evaluators compared rationales, revisited the relevant RDA indicator wording and EURISCO evidence, and agreed on the score best supported by the documented implementation. The independent initial scores were summarized using descriptive measures to assess pre-consensus score convergence. The numerical summary included only cases where all three evaluators had assigned numeric scores on the 1–5 scale. For each included case, the summary calculated whether all evaluators gave the same initial score, the difference between the highest and lowest initial score, and the mean absolute deviation of the three scores from their average. Cases marked as N/A, Removed, or lacking three numeric initial scores were excluded from the numerical summary and retained for qualitative interpretation. The descriptive summary did not determine the final consensus scores.

Qualitative analysis complemented the numerical scoring by drawing on evaluator rationales, consensus notes, and supporting EURISCO evidence to identify implementation patterns and domain-specific considerations affecting how FAIR principles could be applied to EURISCO. For entries with divergent initial scores, the analysis clarified the evidence base for the final consensus score, including how the adapted criteria applied to EURISCO’s infrastructure, data model, and domain-specific context.

## 3 Results and discussion

**Figure 1:**
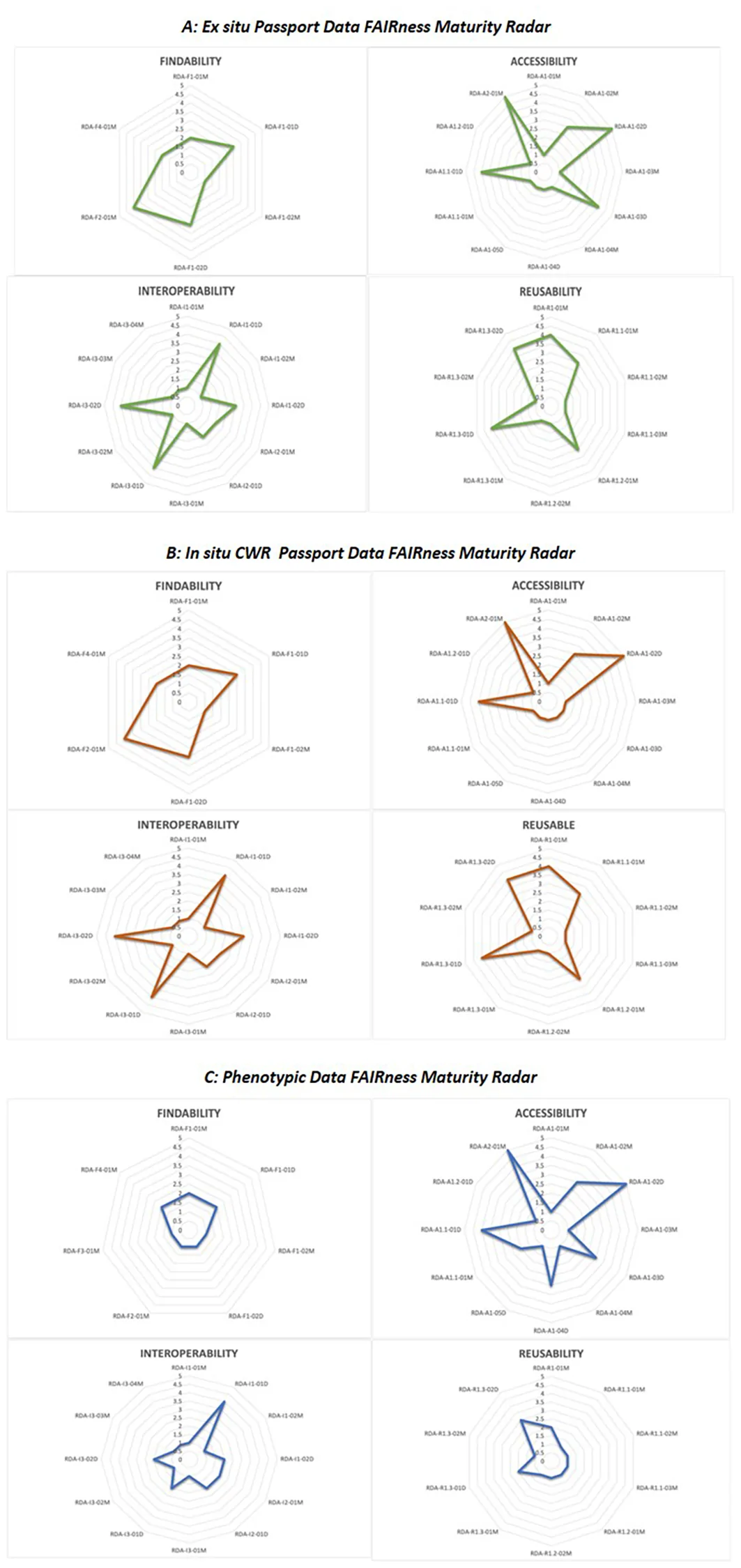
Radar charts illustrating the FAIRness assessment of EURISCO for **(A)** *ex situ* passport, **(B)** *in situ* CWR passport and **(C)** phenotypic data using the RDA FAIR Data Maturity Model/Indicators.

The FAIR assessment employed all 41 indicators from the RDA FAIR Data Maturity Model to three EURISCO data domains: *ex situ* passport data, *in situ* CWR records, and phenotypic datasets (**Figure 1**). The following subsections provide a detailed analysis of the most significant findings, examining each FAIR principle through indicators most relevant to PGR information management and those revealing either substantial strength or notable limitation in current EURISCO implementation. Each indicator discussed is presented with both its descriptive name and formal RDA code. The results provide a basis for broader discussion on the role, feasibility, and impact of FAIR data stewardship in distributed, community-driven infrastructures commonly used in the PGR sector.

### 3.1 Pre-consensus convergence of independent scores

**Supplementary Table 1** provides the full assessment dataset, including the rationale and final consensus score for each indicator within each data domain. Of the 123 indicator-domain entries, (41 indicators x 3 data domains), 118 had complete numeric initial scores from all three evaluators and were included in the descriptive agreement summary (**Table 1**). The remaining five were retained for qualitative interpretation. Among the included entries, 92, or 78%, had identical initial scores from all three evaluators. The overall mean absolute deviation was 0.196. Taken together, these results show that the final consensus scores were based on a high degree of independently aligned scoring, with limited overall dispersion before consensus review.

**Table 1:** Agreement and dispersion of initial evaluator scores.

Data domain	Indicators	Included in numerical summary	Same initial score by all evaluators n (%)	Entries with differing initial scores	Mean absolute deviation
*Ex situ* passport data	41	39	30 (76.9%)	9	0.222
*In situ* CWR passport data	41	39	31 (79.5%)	8	0.165
Phenotypic data	41	40	31 (77.5%)	9	0.200
Total	123	118	92 (78.0%)	26	0.196

Mean absolute deviation is the average distance of the three evaluator scores from their mean; calculated for each entry as $\left(\left|x_{1}-\bar{x}\right|+\left|x_{2}-\bar{x}\right|+\left|x_{3}-\bar{x}\right|\right)/3$, where *x*_1_, *x*_2_, and *x*_3_ are the three initial evaluator scores and $\bar{x}$ is their mean.

Entries with larger score differences were reviewed against the evaluator rationales, EURISCO evidence, and adapted scoring criteria before final consensus scores were assigned. These cases clustered around recurring technical boundary issues where RDA indicators required domain-specific calibration for a PGR information infrastructure, particularly where the evidence crossed boundaries between data and metadata, physical germplasm and digital records, content standards and access protocols, internal persistence and publicly documented persistence, and human-facing access and machine-actionable access. The final consensus scores for these indicators thus reflect evidence-based resolution of specific technical judgments, as opposed to arithmetic aggregation of the independent scores.

### 3.2 EURISCO’s fair strengths and current limitations

#### 3.2.1 Findability

Among its strengths, EURISCO functions as a single, aggregated entry point to an extensive collection of European PGR data that would otherwise be distributed across multiple institutional or national information systems. However, when examined through the lens of the RDA FAIR Maturity Model, several limitations emerge in metadata findability metrics.

Globally unique identifier for metadata (F1-01M) and persistent identifier for metadata (F1-02M): EURISCO does not publish separate metadata records with unique identifiers for each accession. Although EURISCO manages additional metadata to the accession data and uses internal database IDs to ensure persistence in the backend, these identifiers remain hidden from end users and are not globally unique outside the system. There is no publicly available resolving service. As a result, the EURISCO metadata records actually lack globally unique, persistent IDs from an external perspective, which leads to low implementation scores for these FAIR indicators.

Globally unique identifier for data (F1-01D) and persistent identifier for data (F1-02D): Both *ex situ* and *in situ* CWR passport data generally use the composite triplet of institute code, genus, and accession number (INSTCODE:GENUS:ACCENUMB) that is unique within the PGR domain. This identifier system has limitations as it may change over time if, for instance, the genus classification is updated or an institute merges with another. Additionally, about 15% (~300,000) of the accessions have been assigned Digital Object Identifiers (DOIs) (as of March 2025), which are recommended as stable, resolvable, globally unique identifiers for PGR (Garrity et al., 2009; Alercia et al., 2018). However, these DOIs were originally intended to identify the physical germplasm under the Global Information System (GLIS) (https://glis.fao.org/glis/) of the International Treaty on Plant Genetic Resources for Food and Agriculture (ITPGRFA), rather than specifically for data records. EURISCO uses them as proxy identifiers to unambiguously identify the accession’s passport record, but not in the typical manner a data repository would implement a data-specific persistent identifier. Since the focus of DOIs is the physical germplasm and not a versioned dataset, a DOI can only ever be used as an identifier of an exact data object within an information system such as EURISCO. The same DOI can likewise identify datasets in other PGR information systems such as Genesys (https://www.genesys-pgr.org/) or GLIS. Phenotypic data, by contrast, have experiment-specific unique identifiers that enable direct downloads, but limited searchability makes these records difficult to resolve consistently across systems.

Richness of metadata (F2-01M): EURISCO presents a distinctive case in which the standardized passport records describing the PGR effectively function as both the digital object of interest and the descriptive metadata for that physical material. In many conventional data repositories, metadata is conceived as a separate layer documenting dataset-level elements, such as version, creation date, and license, distinct from the underlying data files.Within EURISCO, these elements may remain in the backend if it is deemed non-critical for typical PGR data end-users. Instead, the Multi-Crop Passport Descriptor (MCPD) v2.1 (Alercia et al., 2015) schema captures substantive information and contextual details that allow users to discover, compare, and select germplasm in a highly structured way. While this design diverges from the typical repository model, it nonetheless confers substantial descriptive rigor and, by extension, high metadata richness for discovery and use of passport data within the PGR domain. The same structure similarly explains why *Metadata includes the identifier for the data* (F3-01M) was not scored for the passport-data domains. EURISCO exposes the accession identifier within the passport record itself, without a separate metadata record identifying a distinct data object. Phenotypic data, by contrast, fall significantly short, hampered by the absence of a community-accepted standard for data exchange and documentation. Although EURISCO developed a data exchange template for phenotypic data, it still fails to capture detailed experimental contexts and methodological specifics in a standardized way. This leads to inconsistent documentation of critical contextual metadata, including experimental design details, comprehensive methodology descriptions, standardized trait definitions, and clear provenance of data collection methods, all of which are essential elements for proper discoverability and reuse.

Searchable metadata (F4-01M): While EURISCO offers effective human-facing search interfaces and CSV export functionality for manual data retrieval, it does not currently implement standardized machine-accessible harvesting protocols. External systems cannot automatically query EURISCO for metadata updates, newly added accessions, or bulk metadata retrieval without human intervention. This limitation restricts integration with global aggregators, reduces discoverability by federated search systems, and hinders automated workflows that depend on machine-to-machine metadata exchange. The absence of harvestable metadata endpoints results in low scores for this indicator despite EURISCO’s strong human-accessible search capabilities.

#### 3.2.2 Accessibility

All EURISCO data are openly accessible to users through the web portal, without authentication barriers. Although, accessibility for automated systems remains limited, which creates a gap between human and machine access capabilities.

Data accessible manually (A1-02): All EURISCO datasets can be retrieved via a standard web browser over HTTPS, without any authentication, registration or subscription requirements. The web interface was explicitly designed to serve diverse stakeholders, ranging from genebank managers to researchers to policymakers, ensuring intuitive navigation, flexible search options and straightforward download mechanisms. This open human access model fulfills the requirements of the manual accessibility indicator comprehensively.

Data available through free access protocol (A1.1-01): EURISCO delivers all data through HTTPS, a free, open, universally supported protocol that requires no special permissions or commercial software to implement. The web interface presents data in HTML for viewing and offers downloads in CSV format, both of which are open, non-proprietary formats. This approach ensures that users worldwide, regardless of institutional resources or technical infrastructure, can access EURISCO data without barriers.

Data accessible through persistent protocol (A1-03M/D): These indicators assess whether identifiers resolve persistently to the metadata or data object they identify. EURISCO currently lacks publicly resolvable identifiers for metadata records at the accession level. When users retrieve accession information from EURISCO, they access data through the system’s web interface, which does not generate persistent uniform resource identifiers (URIs) or landing pages that can be reliably cited in publications or linked by external systems. For data identifier resolution, the assessment required a distinction between access to an accession record and persistent identification of a digital data object. Some EURISCO accessions carry DOIs, but these primarily identify physical germplasm material, not a fixed or versioned EURISCO data record. The same DOI may also be used in other PGR information systems, which further limits its role as an identifier for a EURISCO-specific digital object. Thus, while EURISCO supports open access to accession records, it does not yet provide persistent, versioned resolution of accession-level data records in the repository sense expected by these indicators.

Data can be accessed automatically (A1-05D): Despite EURISCO’s strong human accessibility, automated data retrieval remains limited. The catalogue provides public search and export functions, but it does not currently expose a documented application programming interface (API), or equivalent machine-to-machine service for programmatic data retrieval. As a result, bulk data harvesting, real-time synchronization with external systems, automated update notifications, and integration into computational workflows still depend largely on human-facing search and export functions. Breeding API (BrAPI) (Selby et al., 2019), which is already deployed in related breeding and PGR information systems (Selby et al., 2025) is one concrete option for addressing this gap and has been considered in discussions on improving programmatic access to EURISCO. Implementing such an interface would require coordinated changes across National Inventories and National Focal Points and sufficient technical capacity to align existing passport, *in situ* CWR and phenotypic data structures with BrAPI resources while maintaining established validation and update workflows.

Data accessible through an access protocol that supports authentication and authorization (A1.2-01D): This indicator was not included in the final scoring because EURISCO data are provided under an open-access model. Retrieval does not require authentication, authorization, registration, or subscription.

Metadata accessible even when data are no longer available (A2-01): This indicator initially showed considerable divergence in scores because different kinds of evidence could be treated as relevant. One interpretation took the internal retention of EURISCO records as sufficient proof that metadata remain available even when material availability changes. A stricter FAIR interpretation required a publicly documented persistence policy or resolvable record layer that specifies what remains accessible when accession records are withdrawn, corrected, superseded or when institutions cease participation. The consensus review therefore distinguished operational persistence within EURISCO from externally documented metadata persistence. At present, EURISCO does not provide a clearly formulated, publicly communicated policy on metadata retention in such cases. In the absence of explicit persistence guarantees, it remains unclear whether references to specific EURISCO records will continue to resolve to stable metadata descriptions over extended periods.

#### 3.2.3 Interoperability

The interoperability of EURISCO data shows a mix of strengths in using community standards and weaknesses in machine-understandable formatting and linkage.

Data use community-recognized formats (I1-01D): A notable positive pattern is the adherence to well-established data standards for content. The *ex situ* passport data are fully based on MCPD v2.1. This means that field definitions conform to what the PGR community expects. The *in situ* CWR module was designed to align closely with MCPD v2.1 as well. This alignment ensures that both *ex situ* and *in situ* data can be readily understood, validated, and cross-walked to other PGR information systems including the global PGR data aggregator, Genesys. EURISCO thus demonstrates strong performance on this indicator for passport data. Phenotypic data present a different case. As noted previously, phenotypic data currently lack standardization, which further creates interoperability weakness. The EURISCO−specific phenotypic data exchange template ensures a degree of internal consistency, but it reflects a pragmatic compromise and does not fully follow community−wide standards such as the Minimum Information About a Plant Phenotyping Experiment (MIAPPE) (Papoutsoglou et al., 2020). EURISCO adopted this minimum consensus approach primarily for practical reasons. As the network encompasses a diverse range of institutions across multiple countries with varying technical capacities, resources, and existing data management systems, implementing the comprehensive MIAPPE standard would have created an insurmountable barrier to participation for many genebanks. By developing a simplified data exchange template with core required fields, EURISCO prioritized accessibility and broad participation over perfect standardization. This pragmatic strategy equally acknowledges the historical context of genebank data collection. Many institutions have accumulated decades of phenotypic observations following crop-specific descriptor lists, not always within standardized experimental protocols. Nevertheless, this information is of great value for the conservation of PGR. Converting this legacy data to fully MIAPPE-compliant formats would be prohibitively resource-intensive for most contributing institutions. The minimum consensus format offers a workable intermediate solution, allowing valuable phenotypic data to be shared through EURISCO while the community progressively develops capacity for more comprehensive standardization. The high initial divergence for phenotypic data under I1-01D reflected the status of the EURISCO phenotypic data standard as a pragmatic exchange format rather than a fully community-recognized standard comparable to MCPD for passport data or MIAPPE for phenotyping experiments.

Machine-understandable knowledge representation (I1-02M/D): Beyond data schemas, format and technical interoperability aspects reveal gaps for all data domains. EURISCO data can be downloaded as tabular reports (CSV/Excel), which is a widely used format but not a richly structured or machine-actionable knowledge representation. There is no support for semantic web formats, nor a web service to negotiate data format. Data and metadata are not expressed in formal ontologies or linked data format that machines can readily interpret and reason about without explicit human instruction and translation.

Data use community-recognized vocabularies (I2-01D): EURISCO employs controlled vocabularies or code lists in several fields (e.g., ISO 3166 country codes for origin, FAO institute codes for genebanks, MCPD status codes for sample type). These codes are standardized and documented, thus facilitating consistent interpretation and interoperability of those specific attributes. Their role remains largely that of internal reference systems, as they are not exposed as externally resolvable, semantically linked resources. Taxonomy is a case in point, where the genus/species names follow botanical standards, but they are not linked to an external taxonomy database via identifiers. If one system refers to a species by a different synonym, EURISCO has no mechanism to interoperate aside from the name string (Kreide et al., 2019). For phenotypic traits, there is currently an absence of ontology-backed trait definitions in the database, so interoperability of trait data is low, which is a problem that the recommended adoption of ontology-based trait names can possibly address.

Data and Metadata Linked to Related Data and Metadata (I3-01M/D): This particular indicator was generally weak across the EURISCO data domains because the system has been a standalone catalog. There are few explicit references to external datasets or metadata. For example, passport records do not link out to external data like literature, genomic databases, or other repositories. In fact, information about the donor of an accession (donor institute code and number) is a reference to the same germplasm that is available elsewhere. However, these are textual references and not actionable links. In addition, the donor number does not reference a data record, but a physical object. There is no guarantee a user can follow the donor number to an entry in another information system without manual effort. Similarly, *in situ* population records currently exist only in EURISCO, with no links to, for example, biodiversity databases or conservation resources that might also hold information about those populations. For phenotypic data, there is an internal link to passport data (each observation is associated with an accession, enabling users to navigate to the accession’s details, and vice versa). This internal cross-reference is valuable since the phenotypic data references the *ex situ* data by accession identifier, effectively connecting characterization results with germplasm descriptors. It’s not a qualified reference in a semantic web sense, but for users it greatly enhances interoperability between data domains within EURISCO. Functionality for linking phenotypic datasets to peer-reviewed publications has existed for years but remains substantially underutilized, leaving most phenotypic records undocumented regarding their connection to published research.

#### 3.2.4 Reusability

Data are richly described with attributes (R1-01M): EURISCO data records are accompanied by a plurality of relevant attributes that provide context for understanding and reusing the data. *Ex situ* accession records contain core passport information that is crucial for reuse such as taxonomic classification, origin, dates, and other properties describing the material. *In situ* records likewise include essential context for reuse in conservation and research, such as the locality of the wild population, habitat or site name, and responsible management institution. Phenotypic data come with experimental context, albeit minimal, that is vital for reuse of phenotypic information. Each dataset documents where and when an experiment was conducted, what trait was measured and how, and links to which accession was tested. However, much of the information is not structured in a standardized, machine-readable format, often being provided as unstructured plain text or in heterogeneous formats that require significant processing before reuse. Another significant weakness is the limited adoption of existing ontologies or controlled vocabularies for describing traits and methodologies. Despite EURISCO’s efforts to encourage the use of Crop Ontology (CO) (Matteis et al., 2013), implementation remains challenging due to several factors such as (1) the incomplete coverage of available ontologies across the wide diversity of crop species, as trait ontologies have been developed primarily for major crops, leaving many minor and underutilized crops with little or no ontological support, (2) the technical complexity of interpreting and harmonizing trait data to identify the appropriate ontology terms, especially when local descriptors, measurement units, and coding schemes vary widely across sources, (3) and the resource-intensive process of retroactively applying ontologies consistently across large volumes of data, which requires substantial technical capacity and curator time.

Data are released with a clear license (R1.1-01M): EURISCO provides prominently displayed “Terms of Use”, which grant users world-wide, royalty-free, non-exclusive rights to use, reproduce, and redistribute the information for any lawful purpose. The sole requirement imposed on users is the obligation to acknowledge EURISCO as the data source and to follow provided citation guidelines when publishing or publicly presenting analyses based on EURISCO data. These terms function as a de facto open data license and are clearly communicated to human users, but they are expressed in natural language text and do not adopt standard, internationally recognized license frameworks.

Data are released with a standardized license (R1.1-02M): custom licensing language does not conform to widely recognized, standardized license frameworks such as Creative Commons licenses (e.g., CC0, CC-BY, CC-BY-SA). The absence of standardized licensing terminology creates challenges for automated systems: repository aggregators, data catalogs, and research data management platforms cannot programmatically parse EURISCO’s license terms to automatically categorize data according to reuse permissions. Additionally, researchers comparing data from multiple sources may need to individually interpret EURISCO’s custom terms rather than immediately recognizing familiar license categories.

Data follow community standards (R1.3-01D) and data use community metadata standards (R1.3-02D): EURISCO’s data schema aligns with domain-relevant community standards to a large extent, though with differences across data domains. As mentioned, the *ex situ* passport data are fully compliant with MCPD v2.1, and can accordingly be understood and cross-walked to other systems. *In situ* CWR data leverage the same conventions, with a descriptor set designed as an extension of the *ex situ* MCPD format. Phenotypic data, however, require additional alignment with accepted community standards. MIAPPE is not yet fully integrated into EURISCO’s template. Its further integration remains a key objective in ongoing efforts to strengthen EURISCO’s adherence to community best practices.

### 3.3 Practical realities of implementing FAIR in the PGR domain

PGR information systems manage dynamic data linked to heterogeneous, living biological resources. As scientific knowledge advances, taxonomic classifications may be revised, while periodic re-evaluations of plant traits yield additional, often extensive, multifactorial phenotypic data. Duplications or misidentifications are corrected over time, and new phenotypic and genotypic assays are introduced. Moreover, the physical nature of PGR collections necessitates precise correspondence between digital records and the actual biological materials (e.g., seeds, *in vitro* cultures, vegetative propagules or field-maintained accessions) distributed across multiple institutions. The need to maintain tight coupling between digital records and physical specimens is not unique to PGR. Likewise, it arises in museum digitization (Hedrick et al., 2020), biobank sample management (Alkhatib and Gaede, 2024), and geochemical rock repositories (Gard et al., 2019). In genebanking, this requirement carries particularly high operational stakes. Genebank accessions are living collections undergoing continuous biological change, requiring that digital records remain synchronized with physical materials. At the same time, germplasm is distributed across hundreds of autonomous institutions with heterogeneous technical capacities, creating persistent synchronization demands across a dispersed network. Inaccurate or outdated digital records compromise conservation initiatives, undermine research reliability, and jeopardize breeding program success. Collectively, these factors create a multi-layered, multi-modal data ecosystem that is constantly evolving.

EURISCO’s federated architecture adds a governance-driven dimension to data dynamism. Each member state maintains a National Inventory (NI), a comprehensive accession registry overseen by a designated National Inventory Focal Point (NFP). These focal points consolidate (meta)data from every domestic genebank, apply national validation, harmonization and quality-control protocols, and transmit the resulting standardized records to EURISCO. NFPs are explicitly empowered by national mandate to recall or deprecate accession data when material availability changes, e.g., accessions may be withdrawn or flagged as historical when seed viability falls below established thresholds or cannot be successfully regenerated, when new legal or phytosanitary restrictions limit distribution, or when custodial responsibility transfers between institutions. In principle, withdrawn accessions may remain in EURISCO as historical entries to preserve provenance, but in practice, each country exercises its discretion over whether and how to retain or remove such records. Thus, EURISCO’s aggregated data are never static but continuously updated to mirror both scientific revisions and operational realities at the national level. Similar federated platforms, including Genesys (https://www.genesys-pgr.org/), require the same frequent, coordinated (meta)data submissions from distributed custodial sources to maintain accurate, up-to-date information.

Decentralization is both a strength and a constraint for FAIR implementation. Because stewardship resides with sovereign national nodes, EURISCO draws on the capacity of many institutions, which yields broad participation and comprehensive geographic and taxonomic coverage. No single institution could assemble, maintain and continually update a dataset of this scale alone. The same governance model, however, also introduces variability. Countries differ in their policies, technical systems, priorities and institutional capacities. Implementing changes such as adopting new data standards or improving metadata quality therefore requires coordination across the network, since EURISCO cannot unilaterally alter the data supplied by national nodes. FAIR implementation therefore proceeds gradually and iteratively, moving at the pace at which the community can adapt. Some national nodes may adopt new practices quickly, such as assigning DOIs to all accessions, while others may be constrained by limited resources or technical capacity. Consequently, different parts of the EURISCO network may reach different levels of FAIR compliance. Data from one country may be richly annotated and regularly updated, while data from another may remain sparse or infrequently revised. A rigid application of FAIR indicators may therefore produce uneven scores across the network, even where the central aggregator presents the data through a common interface. National sovereignty also means that some data remain subject to national laws and policies, with potential implications for accessibility. For example, a country might choose not to disclose precise location data for rare wild relatives due to conservation concerns. The FAIR principles, being aspirational and not legally binding, must be applied with respect for such policy constraints and negotiated agreements. Within this framework, ECPGR must balance the promotion of open, standardized data sharing with respect for national authority over data stewardship. This federated reality underscores the importance of capacity-building and community consensus in achieving FAIR goals. It is not enough to mandate technical standards. Data curators and providers must be convinced of the benefits and trained in the tools and practices to implement them. Recognizing this, ECPGR has been consistently organizing training workshops and offers ongoing support for NFPs. These initiatives may help build a more uniform capacity across the network, gradually addressing disparities in national contributions. Achieving FAIR in distributed networks depends on each node maintaining reliable data management practices and aligning with shared standards, thereby ensuring coherence and effective integration across the entire network.

A number of initiatives have proposed federated data-sharing solutions that build on FAIR principles. The FLAIR-GG platform, for example, FAIRifies germplasm databases for CWR and wild species in Spain by applying shared metadata standards, and semantic technologies to represent local genebank data in a common framework, exposing them through a federated virtual platform (Cámara Ballesteros et al., 2024). Germplasm data in this setting remain distributed across custodial institutions, recorded in heterogeneous local formats and updated through locally governed curatorial and legal processes. The FLAIR-GG architecture exposes the practical consequence of this reality. Semantic interoperability and federated discovery depend on the completeness and consistency of local records, on stable mappings between local schemas and shared models, on sustained vocabulary governance and on sufficient institutional capacity to maintain transformation workflows as underlying data systems evolve. Where these conditions are weak, the federated layer propagates local deficiencies instead of correcting them (Mesibov, 2018), and FAIR compliance remains a moving target rather than a state that can be enforced centrally (Wilkinson et al., 2019).

Comparable dependencies are also evident in other PGR, biological and collection-based data aggregators operating at a much bigger scale. Genesys provides dataset-level persistent identifiers and per-accession metadata completeness scores through the Passport Data Completeness Index (PDCI) (van Hintum et al., 2011) and exposes MCPD-compliant passport data through standardized BrAPI germplasm endpoints. Phenotypic data in Genesys are nonetheless submitted and managed as heterogeneous tabular files with descriptors defined at the genebank or crop level in the absence of a shared semantic schema. While standard crop descriptors exist, semantic interoperability remains limited because these descriptors are frequently adapted locally and phenotypic data are captured in heterogeneous tables and descriptor meanings remain in institute-specific documentation instead of being consistently aligned to shared, machine−readable vocabularies. Metadata completeness also varies markedly among contributing institutions. Meanwhile, the Global Biodiversity Information Facility (GBIF) (https://www.gbif.org/) provides global occurrence discovery through Darwin Core-based metadata and machine-readable licensing, yet automated processing pipelines still introduce name alterations in up to one in five records and residual errors persist across taxonomy, dates and locality fields (Chapman, 2005; Maldonado et al., 2015; Mesibov, 2018), indicating that FAIR-oriented aggregation cannot fully compensate for inconsistent source data or incomplete community standards. The Distributed System of Scientific Collections (DiSSCo) (https://www.dissco.eu/) is architecturally grounded in FAIR Digital Objects and persistent identifiers for digital specimens, but its conceptual design blueprint confronts a digitization and transcription backlog of approximately 1.5 billion specimens held across more than 130 institutions in 21 countries, and characterizes the persistent difficulties in harmonizing vocabularies, identifiers and provenance practices across this network as organizational and governance challenges (Koureas et al., 2018; Hardisty et al., 2020). Achieving FAIRness is therefore as much a social and institutional challenge as a technical one. The success of any federated infrastructure depends not only on its design, but also on its alignment with the diverse policies, capacities and priorities of participating institutions. When these dimensions are not sufficiently considered, there is a risk that even well−developed platforms may fall short in operational use. New standards and tools, however innovative or technically robust, require local buy−in, sustained support, and the capacity of institutions to integrate them into day−to−day workflows. Without this foundation, technical interoperability alone cannot guarantee meaningful or durable adoption.

A further consideration when implementing FAIR for PGR data is the challenge of legacy data integration. Most PGR collections have accumulated decades of historical information that predates current data standards and the FAIR framework itself. This legacy data often has incomplete metadata, exists in outdated formats, contains inconsistent taxonomic nomenclature, lacks standardized descriptors, or may be incompletely digitized. Collection data recorded in field books from the 1950s, phenotypic data in standalone spreadsheets, or accession information in obsolete database systems all require substantial curation efforts to align with FAIR standards. From a FAIR perspective, incomplete or non-normalized data can reduce reusability and interoperability. EURISCO has improved this over time by encouraging data updates and providing tools like taxonomy mapping to clean up inconsistencies. Nevertheless, the retrospective enhancement of metadata for thousands of existing accessions demands significant resources and expertise. Many institutions face difficult decisions about prioritizing which legacy data to upgrade first, balancing completeness against feasibility. Likewise, historical provenance information may be incomplete by modern standards, limiting the potential to achieve full FAIR compliance for older accessions. This points to a systemic challenge that FAIR compliance is only as good as the source data quality in many cases. In EURISCO, where data quality varies across national nodes, these legacy data challenges can create uneven FAIR implementation, with newer accessions generally having more complete, standards-compliant data than historical entries. Despite these difficulties, incorporating legacy data is essential as these older accessions often represent irreplaceable genetic diversity and valuable historical collecting efforts that must remain discoverable and usable within modern information systems.

In addition to technical infrastructure and institutional mandates, the broader landscape in which PGR data is managed and shared is shaped by intersecting international legal frameworks that introduce both obligations and constraints. The ITPGRFA promotes transparency and data exchange for accessions included in the Multilateral System (MLS) (FAO, 2001), yet this often requires precise documentation of origin, legal status, and use conditions, information that is not always complete or readily available in legacy collections. At the same time, the Nagoya Protocol (CBD, 2010) and its access and benefit-sharing (ABS) framework operationalize national sovereignty over genetic resources by requiring prior informed consent (PIC) and mutually agreed terms (MAT) for access and use of those resources. While the Protocol does not directly govern data, concerns about its interpretation, especially in relation to sensitive materials (e.g., CWR with restricted access or conservation value, accessions with unclear legal status) or associated traditional knowledge, can influence decisions about whether to share related datasets. Within the European Union, Regulation (EU) No 511/2014 harmonizes certain user-compliance obligations for the Nagoya Protocol but leaves Member States free to decide whether and how to regulate access to their own genetic resources. This has produced contrasting national regimes and, in turn, divergent institutional approaches to data disclosure. In the case of Germany, which has not adopted specific ABS access regulations for German genetic resources, access is not conditioned on Nagoya-style PIC and MAT, although other public- and private-law restrictions may still apply (Greiber, 2019). By contrast, France has established a national ABS system under Law No. 2016–1087 and its implementing measures, which introduce declaration and authorization procedures for access to French genetic resources and associated traditional knowledge, while Spain’s Royal Decree 429/2020 sets up an authorization regime for access to plant genetic resources for food and agriculture and certain cultivated resources in line with the ITPGRFA and the Nagoya Protocol (Humphries et al., 2021). In such contexts, genebanks must clarify the ABS status of materials and intended uses, and this due-diligence process often extends to cautious institutional policies on data publication. Entire sets of accession-level information may remain unpublished where compliance requirements are unclear, benefit-sharing arrangements are unresolved, or there is no legal guidance on whether disclosure might enable future claims or liability. These risk-averse decisions, combined with uneven legal guidance, contribute to a patchy and uneven data landscape. Addressing these constraints requires clearer guidance, legal certainty, and ongoing dialogue among policy-makers, data providers, and users to ensure that PGR data can be made available in ways that are responsible, trusted, and widely usable.

It is also important to note that the FAIR maturity indicators themselves continue to evolve, reflecting the diverse data types and domains they serve. For example, the RDA’s FAIR Data Maturity Model recognizes that one size does not fit all. It provides a set of indicators but also allows conditional interpretations tailored to different dataset scopes (Bahim et al., 2020). FAIR implementation remains an iterative, negotiated process, especially in a domain as richly layered and continuously evolving as PGR. This evolving landscape is further complicated by rapidly changing technology and shifting incentive structures. Each technological advance offers new opportunities for enhancing FAIR compliance but also demands continuous learning and system updates (Aguilar Gómez and Bernal, 2023).

### 3.4 Is FAIRness everything?

FAIR principles are invaluable for improving data management, though their limitations and the need for complementary approaches should also be recognized, depending on the practical realities of certain domains. For some disciplines, pursuing “100% FAIRness”, i.e., completely meeting every FAIR criterion to its maximum extent, is neither realistic nor necessarily optimal for institutional resources allocated to research information systems. The FAIR guidelines themselves were conceived as high-level principles rather than rigid standards (Wilkinson et al., 2016). Striving for maximal FAIRness is worthwhile, but it must be balanced against context-specific constraints and the diminishing returns of tackling the most intricate indicators (Mons et al., 2017).

In practice, organizations need to weigh the benefits of comprehensive FAIR compliance against the effort required. For instance, achieving far-reaching interoperability by linking datasets to numerous ontologies and external resources requires careful consideration of whether such connections genuinely enhance data utility for the relevant research community. This motivates the concept of “FAIR enough,” a pragmatic approach that prioritizes essential discoverability and reusability requirements without overemphasizing peripheral metrics (Chu et al., 2024).

While FAIR principles establish essential standards for infrastructure and (meta)data, they are necessarily complementary to, not substitutes for, domain-specific quality validation. The reusability principle fundamentally assumes that data can serve future research contexts, yet this assumption holds only when underlying data accuracy and appropriateness are assured. In the context of EURISCO, comprehensive metadata standardization and persistent identifiers, which are hallmarks of FAIR compliance, create necessary conditions for discovery and access but do not themselves validate the trustworthiness of the germplasm information they describe. Misidentified accessions, inaccurately documented passport data, or poorly understood provenance undermine reusability, even when records are technically highly FAIR, as latent errors can severely limit a dataset’s actual utility. Systematic reuse of data from diverse sources can also introduce hidden biases or errors if the original context is not understood. Boeckhout et al. (2018) pointed out that while FAIR encourages transparency in methodology and metadata, combining datasets without caution can propagate biases or lead to misinterpretation, especially when data of varying provenance and quality are merged. Therefore, fitness-for-purpose and data quality control are critical complements to FAIR. EURISCO’s ongoing data cleaning efforts and validation checks are as important as its FAIR improvements, because without trustworthy content, being findable or accessible has little value. In this vein, at the repository level, the TRUST Principles (Transparency, Responsibility, User focus, Sustainability, and Technology) (Lin et al., 2020), which underscore robust governance, stable infrastructure, and user-centered approaches, complement FAIR’s emphasis on discoverability and reusability by ensuring data remains credible and well-managed over time.

On another note, although FAIR explicitly requires clear licenses for data reuse, it insufficiently accounts for broader legal and ethical constraints in data sharing. For PGR data, intellectual property and sovereign rights may impose usage conditions. While EURISCO primarily contains non-sensitive descriptor data for publicly available accessions, potential inclusion of traditional knowledge or farmer-contributed data requires careful consideration. FAIR acknowledges this nuance by advocating data should be “as open as possible, as closed as necessary”, recognizing that full openness is not mandatory provided access restrictions are documented transparently. The CARE principles (Collective Benefit, Authority to Control, Responsibility, Ethics) complement FAIR by specifically addressing indigenous data governance concerns (Carroll et al., 2021). These principles are especially relevant when managing traditional knowledge associated with PGR, emphasizing indigenous peoples’ rights to control data about their resources and knowledge systems. Implementing both FAIR and CARE frameworks provides a more comprehensive approach to ethically managing culturally sensitive PGR data.

Finally, while FAIR is widely recognized as a foundational principle in modern data management, it must be viewed within the broader landscape of data stewardship and with a balanced perspective that both underscores FAIR’s importance and acknowledges other critical considerations (Jacobsen et al., 2020). As EURISCO’s experience shows, pursuing FAIR principles has led to significant advances, such as standardization, that benefit the broader community. Absolute FAIRness nevertheless remains more of a guiding ideal than a fixed endpoint. In actual use, certain trade-offs are unavoidable. A common example is that a simpler, widely comprehensible data format may be chosen over a semantically rich version that few can parse, emphasizing practical interoperability over exhaustive formal compliance. Furthermore, the costs and effort required to maximize FAIRness must be balanced against tangible benefits (Alharbi et al., 2021). Studies have noted that the final stages of FAIR, particularly full interoperability and reusability, are often the most difficult, sometimes involving hand-crafting complex data structures and extensive curation (Koers et al., 2020; Cámara Ballesteros et al., 2024). Not every information system has the capacity for such large-scale efforts, nor is it always necessary. In many cases, a more agile, iterative approach to FAIR yields greater impact, that is to implement the functionalities most needed by users first (e.g., basic search tools and metadata, which address findability and accessibility), then progressively enhance interoperability and reusability features according to demand and available resources. An integrated outlook recognizes that striving for adequate FAIRness alongside reliability and relevance can be more beneficial than achieving a narrow vision of perfect FAIR compliance. Ultimately, this balanced approach better serves both scientific and societal interests, ensuring data are not only well-managed by technical standards but also truly valuable and usable in real-world contexts. As Aguilar Gomez and Bernal (2023) aptly put it, “FAIR data is not a binary state, and not equal to open or perfect. What matters is making data as FAIR as appropriate for its purpose”.

## 4 Conclusion

The FAIR assessment of EURISCO highlighted that the catalog has made significant strides in making PGR information findable and accessible to humans, while work remains to fully meet interoperability and reusability best practices in machine-actionable ways. In particular, EURISCO’s strengths lie in its unified portal approach, aggregating millions of accession records from across Europe into a single, searchable system. This consolidation inherently improved findability and accessibility compared to the previously far more fragmented PGR data landscape. Notably, EURISCO’s strength is grounded in its network of member countries, effectively functioning as a network-of-networks. By bringing together NI under a shared framework, EURISCO ensures broader coverage, promotes consistent data sharing, and enhances the reliability, comprehensiveness, and sustainability of the information it provides. Likewise, the information system’s adoption of MCPD is a strong foundation that ensures basic interoperability and understanding of the data, while the use of DOI for accessions further enhances global findability and citation capabilities. EURISCO’s data is openly available, which is crucial for reuse, and the information content is of high value and relevance for the PGR community. Nonetheless, limitations in EURISCO’s technical infrastructure, most notably programmatic access through standardized APIs, automated machine-to-machine interaction capabilities, and implementation of semantic web technologies, highlight opportunities to enhance data discoverability and integration across systems and subsequently increase FAIR compliance.

Critically, this paper highlights that the application of FAIR principles must be understood not only as a technical objective but as a process shaped by domain-specific structures, priorities, and constraints. In the domain of PGR, the diversity of biological materials, documentation practices, institutional capacities and regulatory conditions across collections makes uniform implementation inherently complex. Full compliance with every FAIR metric may prove unrealistic or even counterproductive if pursued without regard to local constraints and practical relevance. Overemphasizing technical completeness can divert attention from equally pressing concerns such as data quality, curator capacity, governance structure, and fitness-for-purpose. In this perspective, FAIRness is best evaluated in terms of its contribution to these core functions, where a context-sensitive, intentional approach may yield greater benefit than rigid adherence to every indicator.

EURISCO illustrates that substantial scientific value can be delivered even when certain technical FAIR metrics remain under development, provided that user requirements are effectively addressed. The system’s primary function as a gateway that facilitates access to physical PGR materials maintained across distributed genetic resource centers represents its most fundamental contribution, enabling users to locate relevant germplasm regardless of its physical location. Its FAIRness shortfalls must thereupon be viewed within the broader PGR context, i.e., a long-established, distributed network rooted in biological collections and shaped by decades of human-centered data stewardship, varying national priorities and uneven capacities across systems.

As PGR information systems continue to evolve, EURISCO stands as a pillar in the emerging network-of-networks for PGR data, linking European NI with global data exchange efforts. Its commitment to continuous refinement and system enhancement aligns well with the evolving demands of the PGR community, ensuring continued support for both conservation efforts and crop improvement programs worldwide.

## Data Availability

Publicly available datasets were analyzed in this study. This data can be found here: https://www.ecpgr.org/eurisco/.

## Supplementary material

**Supplementary Table 1** documents the mapping between the RDA FAIR Data Maturity Model indicators and the concrete assessment steps applied to EURISCO’s main data domains (*ex situ* passport, *in situ* crop wild relative passport, and phenotypic data). For each indicator, the table specifies which EURISCO fields, metadata elements, or technical functions were inspected, and how evidence for compliance was classified. This clarifies how high-level FAIR principles were turned into concrete evaluation criteria for a large, federated PGR information system.

## References

[B1] Aguilar Gómez F. Bernal I. (2023). FAIR EVA: Bringing institutional multidisciplinary repositories into the FAIR picture. Sci. Data 10, 764. doi: 10.1038/s41597-023-02652-8 37925573 PMC10625599

[B2] Alercia A. Diulgheroff S. Mackay M. (2015). FAO/Bioversity Multi-Crop Passport Descriptors V. 2.1 [MCPD V.2.1]-December 2015 (Rome: Food and Agriculture Organization of the United Nations and Bioversity International). Available online at: https://cgspace.cgiar.org/handle/10568/69166 (Accessed February 18, 2025).

[B3] Alercia A. López F. M. Hamilton N. R. S. Marsella M. (2018). Digital Object Identifiers for Food Crops: Descriptors and Guidelines of the Global Information System (Rome, Italy: Food and Agriculture Organization of the United Nations). Available online at: https://www.fao.org/3/I8840EN/i8840en.pdf (Accessed February 18, 2025).

[B4] Alharbi E. Skeva R. Juty N. Jay C. Goble C. (2021). Exploring the current practices, costs and benefits of FAIR implementation in pharmaceutical research and development: A qualitative interview study. Data Intell. 3, 507–527. doi: 10.1162/dint_a_00109

[B5] Alkhatib R. Gaede K. I. (2024). Data management in biobanking: Strategies, challenges, and future directions. Biotech. 13, 34. doi: 10.3390/biotech13030034 39311336 PMC11417763

[B6] Anglin N. L. Amri A. Kehel Z. Ellis D. (2018). A case of need: Linking traits to genebank accessions. Biopreserv. Biobanking 16, 337–349. doi: 10.1089/bio.2018.0033 30325668 PMC6204556

[B7] Arita M. Karsch-Mizrachi I. Cochrane G. (2021). The international nucleotide sequence database collaboration. Nucleic Acids Res. 49, D121–D124. doi: 10.1093/nar/gkaa967 33166387 PMC7778961

[B8] Arnaud E. Laporte M. A. Kim S. Aubert C. Leonelli S. Miro B. . (2020). The ontologies community of practice: A CGIAR initiative for big data in agrifood systems. Patterns 1, 100105. doi: 10.1016/j.patter.2020.100105 33205138 PMC7660444

[B9] Convention on Biological Diversity (2010). Nagoya Protocol on Access to Genetic Resources and the Fair and Equitable Sharing of Benefits Arising from Their Utilization to the Convention on Biological Diversity (New York, NY: United Nations), 3008, 3.

[B10] Bahim C. Casorrán-Amilburu C. Dekkers M. Herczog E. Loozen N. Repanas K. . (2020). The FAIR data maturity model: An approach to harmonise FAIR assessments. Data Sci. J. 19, 41. doi: 10.5334/dsj-2020-041

[B11] Boeckhout M. Zielhuis G. A. Bredenoord A. L. (2018). The FAIR guiding principles for data stewardship: Fair enough? Eur. J. Hum. Genet. 26, 931–936. doi: 10.1038/s41431-018-0160-0 29777206 PMC6018669

[B12] Cámara Ballesteros A. Aguayo Jara E. Verykaki E. S. Pastor del Olmo G. Moreno Vazquez S. Torres E. . (2024). The FLAIR-GG federated network of FAIR germplasm data resources. Sci. Data 11, 1386. doi: 10.1038/s41597-024-04243-7 39695186 PMC11655563

[B13] Carroll S. R. Herczog E. Hudson M. Russell K. Stall S. (2021). Operationalizing the CARE and FAIR principles for Indigenous data futures. Sci. Data 8, 108. doi: 10.1038/s41597-021-00892-0 33863927 PMC8052430

[B14] Chamanara J. Gaikwad J. Gerlach R. Algergawy A. Ostrowski A. König-Ries B. (2021). BEXIS2: A FAIR-aligned data management system for biodiversity, ecology and environmental data. Biodivers. Data J. 9, e72901. doi: 10.3897/BDJ.9.e72901 34785977 PMC8589773

[B15] Chapman A. D. (2005). Principles and methods of data cleaning: Primary species and species-occurrence data, version 1.0 Report for the Global Biodiversity Information Facility (Copenhagen: Global Biodiversity Information Facility (GBIF)). Available online at: http://www.gbif.org/document/80528 (Accessed July 8, 2026).

[B16] Chu I. Miller R. Mathews I. Vala A. Sept L. O'Hara R. . (2024). FAIR enough: Building an academic data ecosystem to make real-world data available for translational research. J. Clin. Transl. Sci. 8, e92. doi: 10.1017/cts.2024.530 38836249 PMC11148826

[B17] European Commission (2017). ( EOSC Declaration). Available online at: https://ec.europa.eu/research/openscience/pdf/eosc_declaration.pdf (Accessed February 18, 2025).

[B18] European Commission Expert Group on FAIR Data (2018). Turning FAIR into reality: Final report and action plan from the European Commission Expert Group on FAIR Data (Brussels: Directorate-General for Research and Innovation (European Commission). doi: 10.2777/1524

[B19] FAO . (2001). International Treaty on Plant Genetic Resources for Food and Agriculture (Rome: Food and Agriculture Organization of the United Nations).

[B20] Farley J. Walker D. Geffert B. Chandler N. Eisel L. Friedberg M. . (2024). Creating a transnational green knowledge commons for a socially just sustainability transition. Sustainability 16, 7476. doi: 10.3390/su16177476 30654563

[B21] Gard M. Hasterok D. Halpin J. A. (2019). Global whole-rock geochemical database compilation. Earth Syst. Sci. Data 11, 1553–1566. doi: 10.5194/essd-11-1553-2019

[B22] Garrity G. M. Thompson L. M. Ussery D. W. Paskin N. Baker D. Desmeth P. . (2009). Studies on monitoring and tracking genetic resources: An executive summary. Stand. Genomic Sci. 1, 78–86. doi: 10.4056/sigs.1491 21304641 PMC3035216

[B23] Greiber T. (2019). Implementation of the nagoya protocol in the european union and in Germany. Phytomedicine 53, 313–318. doi: 10.1016/j.phymed.2018.10.020 30392747

[B24] Halewood M. Chiurugwi T. Sackville Hamilton R. Kurtz B. Marden E. Welch E. . (2018). Plant genetic resources for food and agriculture: Opportunities and challenges emerging from the science and information technology revolution. New Phytol. 217, 1407–1419. doi: 10.1111/nph.14993 29359808

[B25] Hardisty A. Saarenmaa H. Casino A. Dillen M. Gödderz K. Groom Q. . (2020). Conceptual design blueprint for the DiSSCo digitization infrastructure - DELIVERABLE D8.1. Res. Ideas Outcomes 6, e54280. doi: 10.3897/rio.6.e54280

[B26] Hedrick B. P. Heberling J. M. Meineke E. K. Turner K. G. Grassa C. J. Park D. S. . (2020). Digitization and the future of natural history collections. BioScience 70, 243–251. doi: 10.1093/biosci/biz163. Available online at: https://www.jstor.org/stable/26923846 (Accessed November 12, 2025).

[B27] Hess C. Ostrom E. (2005). “ A framework for analyzing the knowledge commons,” in understanding knowledge as a commons: From theory to practice, (Cambridge, MA: MIT Press). 21. Available online at: https://surface.syr.edu/sul/21 (Accessed February 1, 2025). Librarians Publications.

[B28] Humphries F. Laird S. Wynberg R. Morrison C. Lawson C. Kolesnikova A. (2021). Survey of access and benefit-sharing country measures accommodating the distinctive features of genetic resources for food and agriculture and associated traditional knowledge. Rome FAO behalf Commission Genet. Resour. For. Food. Agric 92. doi: 10.4060/cb6525en

[B29] Jacobsen A. Azevedo R. M. Juty N. Batista D. Coles S. Cornet R. . (2020). FAIR principles: Interpretations and implementation considerations. Data Intell. 2, 10–29. doi: 10.1162/dint_r_00024

[B30] Karsch-Mizrachi I. Arita M. Burdett T. Cochrane G. Nakamura Y. Pruitt K. D. . (2025). The international nucleotide sequence database collaboration (INSDC): Enhancing global participation. Nucleic Acids Res. 53, D62–D66. doi: 10.1093/nar/gkae1058 39535044 PMC11701530

[B31] Koers H. Bangert D. Hermans E. van Horik R. de Jong M. Mokrane M. (2020). Recommendations for services in a FAIR data ecosystem. Patterns 1, 100058. doi: 10.1016/j.patter.2020.100058 33205119 PMC7660419

[B32] Kotni P. van Hintum T. Maggioni L. Oppermann M. Weise S. (2023). EURISCO update 2023: The European Search Catalogue for Plant Genetic Resources, a pillar for documentation of genebank material. Nucleic Acids Res. 51, D1465–D1469. doi: 10.1093/nar/gkac852 36189883 PMC9825528

[B33] Koureas D. Addink W. Hardisty A. (2018). Digital object cloud for linking natural science collections information; The case of DiSSCo. Biodivers. Inf. Sci. Stand. 2, e25474. doi: 10.3897/biss.2.25474

[B34] Kreide S. Oppermann M. Weise S. (2019). Advancement of taxonomic searches in the European search catalogue for plant genetic resources. Plant Genet. Resour. 17, 559–561. doi: 10.1017/S1479262119000339 41292463

[B35] Kumar P. Hendriks T. Panoutsopoulos H. Brewster C. (2024). Investigating FAIR data principles compliance in horizon 2020 funded Agri-food and rural development multi-actor projects. Agric. Syst. 214, 103822. doi: 10.1016/j.agsy.2023.103822 38826717

[B36] Lin D. Crabtree J. Dillo I. Downs R. R. Edmunds R. Giaretta D. . (2020). The TRUST principles for digital repositories. Sci. Data 7, 144. doi: 10.1038/s41597-020-0486-7 32409645 PMC7224370

[B37] Maldonado C. Molina C. I. Zizka A. Persson C. Taylor C. M. Albán J. . (2015). Estimating species diversity and distribution in the era of big data: To what extent can we trust public databases? Global Ecol. Biogeography A. J. Macroecology 24, 973–984. doi: 10.1111/geb.12326 27656106 PMC5012125

[B38] Mandeville C. P. Koch W. Nilsen E. B. Finstad A. G. (2021). Open data practices among users of primary biodiversity data. BioScience 71, 1128–1147. doi: 10.1093/biosci/biab072 34733117 PMC8560312

[B39] Matteis L. Chibon P. Y. Espinosa H. Skofic M. Finkers R. Bruskiewich R. . (2013). “ Crop ontology: Vocabulary for crop-related concepts”, in: Proceedings of the First International Workshop on Semantics for Biodiversity, (Aachen: CEUR Workshop Proceedings). 979. Available online at: https://ceur-ws.org/Vol-979/WS_s4biodiv2013_paper_4.pdf (Accessed March 5, 2025).

[B40] Mesibov R. (2018). An audit of some processing effects in aggregated occurrence records. ZooKeys 751, 129–146. doi: 10.3897/zookeys.751.24791 29713234 PMC5923217

[B41] Mons B. Cameron N. Velterop J. Dumontier M. da Silva Santos L. O. B. Wilkinson M. D. (2017). Cloudy, increasingly FAIR; Revisiting the FAIR data guiding principles for the European Open Science Cloud. Inf. Serv. Use 37, 49–56. doi: 10.3233/ISU-170824

[B42] Osimo D. Pizzamigliio A. (2023). Creating public sector value through the use of open data. Insights and recommendations from the data.europa.eu campaign. Eur. Commission Directorate-General Commun. Networks Content Technol 28. doi: 10.2830/565958 40793605

[B43] Papoutsoglou E. A. Faria D. Arend D. Arnaud E. Athanasiadis I. N. Chaves I. . (2020). Enabling reusability of plant phenomic datasets with MIAPPE 1.1. New Phytol. 227, 260–273. doi: 10.1111/nph.16544 32171029 PMC7317793

[B44] Pathirana R. Carimi F. (2022). Management and utilization of plant genetic resources for a sustainable agriculture. Plants 11, 2038. doi: 10.3390/plants11152038 35956515 PMC9370719

[B45] Pessanha Santos N. (2023). The expansion of data science: Dataset standardization. Standards 3, 400–410. doi: 10.3390/standards3040028 30654563

[B46] Selby P. Abbeloos R. Adam-Blondon A. F. Agosto-Pérez F. J. Alaux M. Alic I. . (2025). BrAPI v2: Real-world applications for data integration and collaboration in the breeding and genetics community. Database J. Biol. Database Curation 2025, baaf048. doi: 10.1093/database/baaf048 40996713 PMC12462623

[B47] Selby P. Abbeloos R. Backlund J. E. Basterrechea Salido M. Bauchet G. Benites-Alfaro O. E. . (2019). BrAPI-an application programming interface for plant breeding applications. Bioinf. (Oxford England) 35, 4147–4155. doi: 10.1093/bioinformatics/btz190 30903186 PMC6792114

[B48] Taylor L. (2016). The ethics of big data as a public good: Which public? Whose good? Philos. Trans. R. Soc. A. 374, 20160126. doi: 10.1098/rsta.2016.0126 28336800 PMC5124068

[B49] Ugochukwu A. I. Phillips P. W. B. (2024). Open data ownership and sharing: Challenges and opportunities for application of FAIR principles and a checklist for data managers. J. Agric. Food Res. 16, 101157. doi: 10.1016/j.jafr.2024.101157 38826717

[B50] van Hintum T. Engels J. M. M. Maggioni L. (2021). AEGIS, the virtual European genebank: Why it is such a good idea, why it is not working and how it could be improved. Plants 10, 2165. doi: 10.3390/plants10102165 34685973 PMC8541258

[B51] van Hintum T. Menting F. van Strien E. (2011). Quality indicators for passport data in ex situ genebanks. Plant Genet. Resour. 9, 478–485. doi: 10.1017/S1479262111000682 41292463

[B52] Volk G. M. Byrne P. F. Coyne C. J. Flint-Garcia S. Reeves P. A. Richards C. (2021). Integrating genomic and phenomic approaches to support plant genetic resources conservation and use. Plants 10, 2260. doi: 10.3390/plants10112260 34834625 PMC8619436

[B53] Weise S. Lohwasser U. Oppermann M. (2020). Document or lose it-on the importance of information management for genetic resources conservation in genebanks. Plants 9, 1050. doi: 10.3390/plants9081050 32824806 PMC7465628

[B54] Weise S. Oppermann M. Maggioni L. van Hintum T. Knüpffer H. (2017). EURISCO: The European search catalogue for plant genetic resources. Nucleic Acids Res. 45, D1003–D1008. doi: 10.1093/nar/gkw755 27580718 PMC5210606

[B55] Wilkinson M. D. Dumontier M. Aalbersberg I. Appleton G. Axton M. Baak A. . (2016). The FAIR guiding principles for scientific data management and stewardship. Sci. Data 3, 160018. doi: 10.1038/sdata.2016.18 26978244 PMC4792175

[B56] Wilkinson M. D. Dumontier M. Sansone S. A. Bonino da Silva Santos L. O. Prieto M. Batista D. . (2019). Evaluating FAIR maturity through a scalable, automated, community-governed framework. Sci. Data 6, 174. doi: 10.1038/s41597-019-0184-5 31541130 PMC6754447

[B57] Yilmaz P. Kottmann R. Field D. Knight R. Cole J. R. Amaral-Zettler L. . (2011). Minimum information about a marker gene sequence (MIMARKS) and minimum information about any (x) sequence (MIxS) specifications. Nat. Biotechnol. 29, 415–420. doi: 10.1038/nbt.1823 21552244 PMC3367316

[B58] Zwölf C. M. Moreau N. (2023). Assessment of the FAIRness of the Virtual Atomic and Molecular Data Centre following the Research Data Alliance evaluation framework. Eur. Phys. J. D. 77, 70. doi: 10.1140/epjd/s10053-023-00649-x

