# Supplementary material for: FAIRness at all costs? Practical limitations in applying the FAIR principles to plant genetic resources data
Source: Front Plant Sci. 2026 Aug 6;17:1888670. doi: 10.3389/fpls.2026.1888670 (PMC13490073; doi:10.3389/fpls.2026.1888670)
Supplement: Supplementary file 1 [file DataSheet1.pdf]

Table S1a. Mapping of RDA FAIR Metrics with Assessment Methodology for EURISCO's *Ex situ* Passport Data (Assessment period: December 2024-February 2025; Final consensus review completed: March 2025)

RDA ID	RDA FAIR Metric		Ex situ				
		Ex Situ Passport Data	Evaluator 1	Evaluator 2	Evaluator 3	FINAL. (Discussed)	Implementation Criteria
**Findability**							
RDA-F1-01M	Metadata is identified by a persistent identifier	System-wide verification of persistent identifier implementation for ex situ passport metadata	4 (metadata exists in the EURISCO backend and has unique and stable identifiers, but is not publicly available); 1 (otherwise)	4	3 (Always from the point of view of the publicly visible possibilities, I would not include the intere or non-explained uniqueness and persistence in the rating)	2 (Basic identifier system exists (e.g., internal database IDs) that provides uniqueness within the system)	5: All metadata records have PIDs with version history and change tracking;<br>4: Majority of metadata records (>75%) have persistent identifiers with global resolution;<br>3: Initial implementation of persistent identifiers for some metadata records (25-75%);<br>2: Basic identifier system exists (e.g., internal database IDs) that provides uniqueness within the system;<br>1: No systematic identification system implemented

RDA-F1-01D	Data is identified by a persistent identifier	System-wide assessment of persistent identifier implementation for ex situ accession data	3 (although the identifiers are intended to identify the physical material, all accession entries have the unique triplet of INSTCODE:GENUS:ACCENUMB + about 15% have a DOI in addition; thus, passport data can be uniquely identified within EURISCO); 1 (passport data can change over time although the accession DOI remains the same + a DOI does not exclusively point to EURISCO)	3	3	3	5: All data has PIDs with version history and bidirectional linkage capabilities;<br>4: Majority of data (>75%) has persistent identifiers with global resolution;<br>3: Initial implementation of persistent identifiers for some data (25-75%);<br>2: Basic identifier system exists (e.g., internal database IDs) that provides uniqueness within the system;<br>1: No systematic identification system implemented

RDA-F1-02M	Metadata is identified by a globally unique identifier	Evaluation of global uniqueness of ex situ metadata identifiers across PGR systems	1 (internal identifiers are unique and persistent within EURISCO, but are running numbers and thus not unique globally)	1	1	1	5: All metadata has globally unique IDs with cross-system resolution;<br>4: All metadata has globally unique identifiers;<br>3: Most metadata has unique identifiers;<br>2: Some unique identification;<br>1: No globally unique identifiers
RDA-F1-02D	Data is identified by a globally unique identifier	Evaluation of global uniqueness of ex situ accession identifiers and cross-referencing	3 (the triplet is globally unique, but can change over time and, most important, identifies the physical object and not the data (which can also change over time)	3	3	3	5: All data has globally unique IDs with cross-system resolution;<br>4: All data has globally unique identifiers;<br>3: Most data has unique identifiers;<br>2: Some unique identification;<br>1: No globally unique identifiers

RDA-F2-01M	Rich metadata is provided to allow discovery	Assessment of MCPD v2.1 compliance with emphasis on conservation status and taxonomy	4	4	4	4	5: Complete domain-specific metadata with extended descriptors;<br>4: Full compliance with relevant standards;<br>3: Most required fields completed;<br>2: Basic metadata only;<br>1: Minimal metadata
RDA-F3-01M	Metadata includes the identifier for the data	Verification of explicit links between passport metadata and accession identifiers	NA (as accession identifiers are themselves part of the data)	1	2 (difficulty: what about linkage to landing pages/ACCURL ?)	Removed	5: Bidirectional linkage with version tracking;<br>4: Clear reference to all data identifiers;<br>3: Most metadata connected to identifiers;<br>2: Basic connection exists;<br>1: No identifier inclusion
RDA-F4-01M	Metadata is offered in such a way that it can be harvested and indexed	Assessment of ex situ data harvesting mechanisms (OAI-PMH, APIs, export formats)	2	2	2	2	5: Multiple harvesting protocols with automated synchronization;<br>4: Standard harvesting mechanisms implemented;<br>3: Basic harvesting capability;<br>2: Limited harvesting support;<br>1: No harvestability
**Accessibility**							

RDA-A1-01M	Metadata contains information to enable the user to get access to the data	Evaluation of access information for germplasm material requests	1	1	1	1	5: Complete access instructions for all resource types; 4: Clear access information provided;<br>3: Basic access information;<br>2: Limited access information;<br>1: No access information
RDA-A1-02M	Metadata can be accessed manually (i.e. with human intervention)	Assessment of human-readable interfaces for ex situ passport data	3	3	3	3	5: Advanced user interfaces with domain-specific features;<br>4: Full manual access implementation;<br>3: Basic manual access capability;<br>2: Limited manual access;<br>1: No manual access
RDA-A1-02D	Data can be accessed manually (i.e. with human intervention)	Assessment of human interfaces for accessing complete ex situ accession data	5	5	5	5	5: Advanced interfaces with data type-specific features;<br>4: Complete manual access implementation;<br>3: Basic manual access capability;<br>2: Limited manual access;<br>1: No manual access

RDA-A1-03M	Metadata identifier resolves to a metadata record	Testing of identifier resolution for ex situ passport metadata	1	1	4 (also not clear for me, because of the dualism of the passport data as metadata and data)	1	5: Direct resolution with content negotiation;<br>4: Reliable resolution to complete records;<br>3: Basic resolution functionality;<br>2: Limited resolution capability;<br>1: No resolution implemented
RDA-A1-03D	Data identifier resolves to a digital object	Testing of identifier resolution for ex situ accession records	4 (if we focus on EURISCO and consider both the triplet and the DOI also to be an identifier of the data)	4	1 (the passport data record itself is not a digital object)	4	5: Direct resolution with content negotiation;<br>4: Reliable resolution to digital objects;<br>3: Basic resolution functionality;<br>2: Limited resolution capability;<br>1: No resolution implemented
RDA-A1-04M	Metadata is accessed through standardised protocol	Evaluation of protocols for accessing ex situ passport metadata	1	1	1	1	5: Multiple standardized protocols with documentation;<br>4: Standard protocol implementation;<br>3: Basic protocol standardization;<br>2: Limited protocol standardization;<br>1: No standardized protocol

RDA-A1-04D	Data is accessible through standardised protocol	Evaluation of protocols for accessing ex situ accession data	1 (just by a web application)	1	4 (MCPD is a standardized protocol, I do not see the technical transport as a criteria)	data retrieval is via HTTPS	5: Multiple standardized protocols with data-type specific endpoints;<br>4: Standard protocol implementation;<br>3: Basic protocol standardization;<br>2: Limited protocol standardization;<br>1: No standardized protocol
RDA-A1-05D	Data can be accessed automatically (i.e. by a computer program)	Assessment of APIs and programmatic access to ex situ data	1	1	1 (by the way: what is with the BRAPI?)	1	5: Comprehensive API with data-type specific query capabilities;<br>4: Full programmatic access implementation;<br>3: Basic programmatic access;<br>2: Limited automatic access;<br>1: No programmatic access
RDA-A1.1-01M	Metadata is accessible through a free access protocol	Verification of free access to ex situ passport metadata	1	1	1	1	5: Multiple free access protocols with documentation; 4: Free access protocol fully implemented; 3: Basic free access implemented; 2: Limited free access; 1: No free access protocol

RDA-A1.1-01D	Data is accessible through a free access protocol	Verification of free access to ex situ accession data	4 (if we consider the web application a free access protocol)	4	3	4	5: Free access with documentation of any limitations;<br>4: Free access protocol fully implemented;<br>3: Basic free access implemented;<br>2: Limited free access;<br>1: No free access protocol
RDA-A1.2-01D	Data is accessible through an access protocol that supports authentication and authorisation	Assessment of authentication for restricted ex situ data (e.g., sensitive landrace data)	1 (all data freely available by definition)	1	N/A (data are free)	1	5: Granular authentication reflecting legal frameworks;<br>4: Full authentication implementation where needed; 3: Basic authentication capability; 2: Limited authentication support; 1: No authentication support
RDA-A2-01M	Metadata is guaranteed to remain available after data is no longer available	Verification that ex situ metadata persists when material is unavailable	5 (yes, in the EURISCO backend)	5	1	5	5: Complete historical preservation with status indicators;<br>4: Full metadata persistence guarantee;<br>3: Basic metadata preservation;<br>2: Limited persistence guarantee;<br>1: No persistence guarantee

**Interoperability**							
RDA-I1-01M	Metadata uses knowledge representation expressed in standardised format	Evaluation of standardized formats (MCPD v2.1) for ex situ passport metadata	1	1	1	1	5: Multiple standardized formats with schema definitions;<br>4: Standardized format fully implemented;<br>3: Basic standardized format used;<br>2: Limited standardization;<br>1: No standardized format
RDA-I1-01D	Data uses knowledge representation expressed in standardised format	Assessment of standardized data formats for ex situ passport data	4	4	4 (here MCPD is to evaluate)	4	5: Multiple standardized formats for different data aspects;<br>4: Standardized format fully implemented;<br>3: Basic standardized format used;<br>2: Limited standardization;<br>1: No standardized format

RDA-I1-02M	Metadata uses machine-understandable knowledge representation	Evaluation of machine-readable formats for ex situ metadata	1	1	1	1	5: Formal semantic representation with domain ontologies;<br>4: Machine-understandable format implemented;<br>3: Basic machine readability;<br>2: Limited machine understanding;<br>1: No machine-understandable representation
RDA-I1-02D	Data uses machine-understandable knowledge representation	Assessment of machine-readable formats for biological status, taxonomy, etc.	3	3	3	3	5: Formal semantic representation of complex relationships;<br>4: Machine-understandable format implemented;<br>3: Basic machine readability;<br>2: Limited machine understanding;<br>1: No machine-understandable representation

RDA-I2-01M	Metadata uses FAIR-compliant vocabularies	Evaluation of controlled vocabularies for biological status, material type, etc.	2	2	2	2	5: Multiple FAIR-compliant vocabularies with documentation;<br>4: FAIR-compliant vocabularies used consistently;<br>3: Basic vocabulary standardization;<br>2: Limited vocabulary compliance;<br>1: No FAIR-compliant vocabularies
RDA-I2-01D	Data uses FAIR-compliant vocabularies	Assessment of FAIR vocabulary usage in passport data fields	2	3	3	data use MCPD fields, ISO country codes, FAO Institute codes.	5: Comprehensive FAIR vocabularies across all aspects;<br>4: FAIR-compliant vocabularies used consistently;<br>3: Basic vocabulary standardization;<br>2: Limited vocabulary compliance;<br>1: No FAIR-compliant vocabularies

RDA-I3-01M	Metadata includes references to other metadata	Verification of references to related metadata (collecting missions, taxonomy)	1	1	1	1	5: Comprehensive references to related metadata; 4: Clear references to other metadata; 3: Basic references implemented; 2: Limited references; 1: No references to other metadata
RDA-I3-01D	Data includes references to other data	Assessment of references to related accessions (duplicates, parent-child)	4	4	4	4	5: Comprehensive references with relationship types;<br>4: Clear references to other data;<br>3: Basic references implemented;<br>2: Limited references;<br>1: No references to other data
RDA-I3-02M	Metadata includes references to other data	Verification that ex situ metadata references characterizatio n data	1	1	1	1	5: Comprehensive references to diverse data types;<br>4: Clear references to related data;<br>3: Basic data references;<br>2: Limited data references;<br>1: No references to data

RDA-I3-02D	Data includes qualified references to other data	Assessment of qualified references in ex situ data (relationship types)	4	4	4	4	5: Comprehensive qualified references with relationship model;<br>4: Qualified references consistently implemented;<br>3: Basic qualified references;<br>2: Limited qualification;<br>1: No qualified references
RDA-I3-03M	Metadata includes qualified references to other metadata	Verification of qualified metadata references in ex situ documentation	1	1	1	1	5: Comprehensive qualified references with relationship types;<br>4: Qualified metadata references implemented;<br>3: Basic qualified references;<br>2: Limited qualification;<br>1: No qualified references
RDA-I3-04M	Metadata include qualified references to other data	Assessment of qualified references from ex situ metadata to diverse data types	1	1	1	1	5: Comprehensive qualified references with specific semantics;<br>4: Qualified data references implemented;<br>3: Basic qualified references;<br>2: Limited qualification;<br>1: No qualified references
**Reusability**							

RDA-R1-01M	Plurality of accurate and relevant attributes are provided to allow reuse	Evaluation of attribute completeness for ex situ reuse (breeding, research)	4	4	4	4	5: Comprehensive attributes across all dimensions;<br>4: Multiple relevant attributes provided;<br>3: Basic reuse attributes;<br>2: Limited attribute plurality;<br>1: Insufficient attributes for reuse
RDA-R1.1-01M	Metadata includes information about the licence under which the data can be reused	Assessment of licensing information for ex situ data and material	1	3	3	whether the EURISCO Terms of Use constitute, or at least function as "information about the license under which the data can be reused." If it fulfills the requirement of specifying a data usage license or reuse terms.	5: Comprehensive licensing for data and material;<br>4: Clear license information provided;<br>3: Basic license information;<br>2: Limited license information;<br>1: No license information

RDA-R1.1-02M	Metadata refers to a standard reuse licence	Verification of standard license references for ex situ data	1	1	1	1 (not a standard CC license or something equally recognized.)	5: References to both data licenses and material agreements;<br>4: Standard license references implemented;<br>3: Basic license references;<br>2: Limited standardization;<br>1: No standard license
RDA-R1.1-03M	Metadata refers to a machine-understandable reuse licence	Evaluation of machine-readable licenses for ex situ data	1	1	1	1	5: Machine-readable licenses for all aspects;<br>4: Machine-understandable license implemented;<br>3: Basic machine-readable elements;<br>2: Limited machine readability;<br>1: No machine-understandable license
RDA-R1.2-01M	Metadata includes provenance information according to community-specific standards	Assessment of conservation history and passport provenance	1 (not in the metadata in the strict sense)	3	2	institute codes, donor info, origin. The date of update, national inventory?	5: Comprehensive provenance following standards;<br>4: Community-standard provenance implemented;<br>3: Basic provenance information;<br>2: Limited provenance;<br>1: No community-specific provenance

RDA-R1.2-02M	Metadata includes provenance information according to a cross-community language	Evaluation of cross-domain provenance for ex situ documentation	1	1	1	1	5: Cross-community provenance with extensions; 4: Cross-community provenance implemented; 3: Basic cross-community elements; 2: Limited cross-community language; 1: No cross-community provenance
RDA-R1.3-01M	Metadata complies with a community standard	Verification of MCPD v2.1 compliance for ex situ passport data	1 (if we focus on the metadata in the EURISCO backend only)	1	1	1	5: Full compliance with multiple standards; 4: Community standard fully implemented; 3: Basic standard compliance; 2: Limited compliance; 1: No community standard compliance
RDA-R1.3-01D	Data complies with a community standard	Assessment of ex situ data compliance with PGR standards	4 (EURISCO-MCPD)	4	4	4	5: Compliance with multiple data-type standards; 4: Community standard fully implemented; 3: Basic standard compliance; 2: Limited compliance; 1: No community standard compliance

RDA-R1.3-02M	Metadata is expressed in compliance with a machine-understandable community standard	Evaluation of machine-readable ex situ metadata standard implementation	1	1	1	1	5: Machine-understandable with validation tools;<br>4: Machine-understandable standard implemented;<br>3: Basic machine-understandable elements;<br>2: Limited machine understanding;<br>1: No machine-understandable standard
RDA-R1.3-02D	Data is expressed in compliance with a machine-understandable community standard	Assessment of machine-readable ex situ data standard compliance	4 (yes, in principle)	4	4	4	5: Machine-understandable with semantic richness;<br>4: Machine-understandable standard implemented;<br>3: Basic machine-understandable elements;<br>2: Limited machine understanding;<br>1: No machine-understandable standard

Table S1b. Mapping of RDA FAIR Metrics with Assessment Methodology for EURISCO's *In situ CWR* Passport Data

RDA ID	RDA FAIR Metric	*In situ* CWR				
		**Evaluator 1**	**Evaluator 2**	**Evaluator 3**	**Final (Discussed)**	**Implementation Criteria**
**Findability**						
RDA-F1-01M	Metadata is identified by a persistent identifier	4 (metadata exists in the EURISCO backend and has unique and stable identifiers, but is not publicly available); 1 (otherwise)	4	3	2	5: All metadata records have PIDs with version history and change tracking;<br>4: Majority of metadata records (>75%) have persistent identifiers with global resolution;<br>3: Initial implementation of persistent identifiers for some metadata records (25-75%);<br>2: Basic identifier system exists (e.g., internal database IDs) that provides uniqueness within the system;<br>1: No systematic identification system implemented

RDA-F1-01D	Data is identified by a persistent identifier	3 (ditto; all populations entries have the unique triplet of INSTCODE:GENUS:ACCENUMB (no DOIs so far); thus, passport data can be uniquely identified within EURISCO); 1 (passport data can change over time although the accession identifier remains the same + an ideitifer does not exclusively point to EURISCO)	3	3	3	5: All data has PIDs with version history and bidirectional linkage capabilities;<br>4: Majority of data (>75%) has persistent identifiers with global resolution;<br>3: Initial implementation of persistent identifiers for some data (25-75%);<br>2: Basic identifier system exists (e.g., internal database IDs) that provides uniqueness within the system;<br>1: No systematic identification system implemented
RDA-F1-02M	Metadata is identified by a globally unique identifier	1 (dito)	1	1	1	5: All metadata has globally unique IDs with cross-system resolution;<br>4: All metadata has globally unique identifiers;<br>3: Most metadata has unique identifiers;<br>2: Some unique identification;<br>1: No globally unique identifiers

RDA-F1-02D	Data is identified by a globally unique identifier	3 (the triplet is globally unique, but can change over time and, most important, identifies the physical object and not the data (which can also change over time)	3	3	3	5: All data has globally unique IDs with cross-system resolution;<br>4: All data has globally unique identifiers;<br>3: Most data has unique identifiers;<br>2: Some unique identification;<br>1: No globally unique identifiers
RDA-F2-01M	Rich metadata is provided to allow discovery	4	4	4	4	5: Complete domain-specific metadata with extended descriptors;<br>4: Full compliance with relevant standards;<br>3: Most required fields completed;<br>2: Basic metadata only;<br>1: Minimal metadata
RDA-F3-01M	Metadata includes the identifier for the data	NA (as accession identifiers are themselves part of the data)	1	N/A	Removed	5: Bidirectional linkage with version tracking;<br>4: Clear reference to all data identifiers;<br>3: Most metadata connected to identifiers;<br>2: Basic connection exists;<br>1: No identifier inclusion

RDA-F4-01M	Metadata is offered in such a way that it can be harvested and indexed	2	2	2	2	5: Multiple harvesting protocols with automated synchronization;<br>4: Standard harvesting mechanisms implemented;<br>3: Basic harvesting capability;<br>2: Limited harvesting support;<br>1: No harvestability
**Accessibility**						
RDA-A1-01M	Metadata contains information to enable the user to get access to the data	1	1	1	1	5: Complete access instructions for all resource types; 4: Clear access information provided;<br>3: Basic access information;<br>2: Limited access information;<br>1: No access information
RDA-A1-02M	Metadata can be accessed manually (i.e. with human intervention)	3	3	3	3	5: Advanced user interfaces with domain-specific features;<br>4: Full manual access implementation;<br>3: Basic manual access capability;<br>2: Limited manual access;<br>1: No manual access
RDA-A1-02D	Data can be accessed manually (i.e. with human intervention)	5	5	5	5	5: Advanced interfaces with data type-specific features;<br>4: Complete manual access implementation;<br>3: Basic manual access capability;<br>2: Limited manual access;<br>1: No manual access

RDA-A1-03M	Metadata identifier resolves to a metadata record	1	1	1	1	5: Direct resolution with content negotiation;<br>4: Reliable resolution to complete records;<br>3: Basic resolution functionality;<br>2: Limited resolution capability;<br>1: No resolution implemented
RDA-A1-03D	Data identifier resolves to a digital object	4 (if we focus on EURISCO and consider both the triplet and the DOI also to be an identifier of the data)	1	1	no universal resolvable PIDs	5: Direct resolution with content negotiation;<br>4: Reliable resolution to digital objects;<br>3: Basic resolution functionality;<br>2: Limited resolution capability;<br>1: No resolution implemented
RDA-A1-04M	Metadata is accessed through standardised protocol	1	1	1		5: Multiple standardized protocols with documentation;<br>4: Standard protocol implementation;<br>3: Basic protocol standardization;<br>2: Limited protocol standardization;<br>1: No standardized protocol

RDA-A1-04D	Data is accessible through standardised protocol	1 (just by a web application)	1	2	data retrieval is via HTTPS	5: Multiple standardized protocols with data-type specific endpoints;<br>4: Standard protocol implementation;<br>3: Basic protocol standardization;<br>2: Limited protocol standardization;<br>1: No standardized protocol
RDA-A1-05D	Data can be accessed automatically (i.e. by a computer program)	1	1	1	1	5: Comprehensive API with data-type specific query capabilities;<br>4: Full programmatic access implementation;<br>3: Basic programmatic access;<br>2: Limited automatic access;<br>1: No programmatic access
RDA-A1.1-01M	Metadata is accessible through a free access protocol	1	1	1	1	5: Multiple free access protocols with documentation; 4: Free access protocol fully implemented; 3: Basic free access implemented; 2: Limited free access; 1: No free access protocol
RDA-A1.1-01D	Data is accessible through a free access protocol	4 (if we consider the web application a free access protocol)	4	3	4	5: Free access with documentation of any limitations;<br>4: Free access protocol fully implemented;<br>3: Basic free access implemented;<br>2: Limited free access;<br>1: No free access protocol

RDA-A1.2-01D	Data is accessible through an access protocol that supports authentication and authorisation	1 (all data freely available by definition)	1	N/A	1 (Not needed. All data is open, might skew the FAIR figure)	5: Granular authentication reflecting legal frameworks; 4: Full authentication implementation where needed; 3: Basic authentication capability; 2: Limited authentication support; 1: No authentication support
RDA-A2-01M	Metadata is guaranteed to remain available after data is no longer available	5 (yes, in the EURISCO backend)	5	1	5	5: Complete historical preservation with status indicators;<br>4: Full metadata persistence guarantee;<br>3: Basic metadata preservation;<br>2: Limited persistence guarantee;<br>1: No persistence guarantee
**Interoperability**						
RDA-I1-01M	Metadata uses knowledge representation expressed in standardised format	1	1	1	1	5: Multiple standardized formats with schema definitions;<br>4: Standardized format fully implemented;<br>3: Basic standardized format used;<br>2: Limited standardization;<br>1: No standardized format

RDA-I1-01D	Data uses knowledge representation expressed in standardised format	4	4	3	4	5: Multiple standardized formats for different data aspects;<br>4: Standardized format fully implemented;<br>3: Basic standardized format used;<br>2: Limited standardization;<br>1: No standardized format
RDA-I1-02M	Metadata uses machine-understandable knowledge representation	1	1	1	1	5: Formal semantic representation with domain ontologies;<br>4: Machine-understandable format implemented;<br>3: Basic machine readability;<br>2: Limited machine understanding;<br>1: No machine-understandable representation
RDA-I1-02D	Data uses machine-understandable knowledge representation	3	3	3	3	5: Formal semantic representation of complex relationships;<br>4: Machine-understandable format implemented;<br>3: Basic machine readability;<br>2: Limited machine understanding;<br>1: No machine-understandable representation

RDA-I2-01M	Metadata uses FAIR-compliant vocabularies	2	2	2	2	5: Multiple FAIR-compliant vocabularies with documentation;<br>4: FAIR-compliant vocabularies used consistently;<br>3: Basic vocabulary standardization;<br>2: Limited vocabulary compliance;<br>1: No FAIR-compliant vocabularies
RDA-I2-01D	Data uses FAIR-compliant vocabularies	2	2	2	2	5: Comprehensive FAIR vocabularies across all aspects;<br>4: FAIR-compliant vocabularies used consistently;<br>3: Basic vocabulary standardization;<br>2: Limited vocabulary compliance;<br>1: No FAIR-compliant vocabularies
RDA-I3-01M	Metadata includes references to other metadata	1	1	1	1	5: Comprehensive references to related metadata; 4: Clear references to other metadata; 3: Basic references implemented; 2: Limited references; 1: No references to other metadata
RDA-I3-01D	Data includes references to other data	4	4	4	4	5: Comprehensive references with relationship types;<br>4: Clear references to other data;<br>3: Basic references implemented;<br>2: Limited references;<br>1: No references to other data

RDA-I3-02M	Metadata includes references to other data	1	1	1	1	5: Comprehensive references to diverse data types;<br>4: Clear references to related data;<br>3: Basic data references;<br>2: Limited data references;<br>1: No references to data
RDA-I3-02D	Data includes qualified references to other data	4	4	4	4	5: Comprehensive qualified references with relationship model;<br>4: Qualified references consistently implemented;<br>3: Basic qualified references;<br>2: Limited qualification;<br>1: No qualified references
RDA-I3-03M	Metadata includes qualified references to other metadata	1	1	1	1	5: Comprehensive qualified references with relationship types;<br>4: Qualified metadata references implemented;<br>3: Basic qualified references;<br>2: Limited qualification;<br>1: No qualified references

RDA-I3-04M	Metadata include qualified references to other data	1	1	1	1	5: Comprehensive qualified references with specific semantics;<br>4: Qualified data references implemented;<br>3: Basic qualified references;<br>2: Limited qualification;<br>1: No qualified references
**Reusability**						
RDA-R1-01M	Plurality of accurate and relevant attributes are provided to allow reuse	4	4	4	4	5: Comprehensive attributes across all dimensions;<br>4: Multiple relevant attributes provided;<br>3: Basic reuse attributes;<br>2: Limited attribute plurality;<br>1: Insufficient attributes for reuse
RDA-R1.1-01M	Metadata includes information about the licence under which the data can be reused	1	3	3		5: Comprehensive licensing for data and material;<br>4: Clear license information provided;<br>3: Basic license information;<br>2: Limited license information;<br>1: No license information

RDA-R1.1-02M	Metadata refers to a standard reuse licence	1	1	1	1	5: References to both data licenses and material agreements;<br>4: Standard license references implemented;<br>3: Basic license references;<br>2: Limited standardization;<br>1: No standard license
RDA-R1.1-03M	Metadata refers to a machine-understandable reuse licence	1	1	1	1	5: Machine-readable licenses for all aspects;<br>4: Machine-understandable license implemented;<br>3: Basic machine-readable elements;<br>2: Limited machine readability;<br>1: No machine-understandable license
RDA-R1.2-01M	Metadata includes provenance information according to community-specific standards	1 (not in the metadata in the strict sense)	3	2		5: Comprehensive provenance following standards;<br>4: Community-standard provenance implemented;<br>3: Basic provenance information;<br>2: Limited provenance;<br>1: No community-specific provenance

RDA-R1.2-02M	Metadata includes provenance information according to a cross-community language	1	1	1	1	5: Cross-community provenance with extensions;<br>4: Cross-community provenance implemented;<br>3: Basic cross-community elements;<br>2: Limited cross-community language;<br>1: No cross-community provenance
RDA-R1.3-01M	Metadata complies with a community standard	1 (if we focus on the metadata in the EURISCO backend only)	1	1	1	5: Full compliance with multiple standards;<br>4: Community standard fully implemented;<br>3: Basic standard compliance;<br>2: Limited compliance;<br>1: No community standard compliance
RDA-R1.3-01D	Data complies with a community standard	4 (MCPD-derived standard agreed upon within ECPGR)	4	4	4	5: Compliance with multiple data-type standards;<br>4: Community standard fully implemented;<br>3: Basic standard compliance;<br>2: Limited compliance;<br>1: No community standard compliance

RDA-R1.3-02M	Metadata is expressed in compliance with a machine-understandable community standard	1	1	1	1	5: Machine-understandable with validation tools;<br>4: Machine-understandable standard implemented;<br>3: Basic machine-understandable elements;<br>2: Limited machine understanding;<br>1: No machine-understandable standard
RDA-R1.3-02D	Data is expressed in compliance with a machine-understandable community standard	4 (yes, in principle)	4	4	4	5: Machine-understandable with semantic richness;<br>4: Machine-understandable standard implemented;<br>3: Basic machine-understandable elements;<br>2: Limited machine understanding;<br>1: No machine-understandable standard

Table S1c. Mapping of RDA FAIR Metrics with Assessment Methodology for EURISCO's Phenotypic Data

RDA ID	RDA FAIR Metric	Phenotypic Data					
		**Evaluator 1**	**Evaluator 2**	**Evaluator 3**	**Final (Discussed)**	**Phenotypic Data/Characterization and Evaluation Data (C&E)**	**Implementation Criteria**
**Findability**							
RDA-F1-01M	Metadata is identified by a persistent identifier	4 (internal metadata as in the case of passport data); 1 (otherwise)	4	1	2	System-wide verification of persistent identifier implementation for C&E metadata	5: All metadata records have PIDs with version history and change tracking;<br>4: Majority of metadata records (>75%) have persistent identifiers with global resolution;<br>3: Initial implementation of persistent identifiers for some metadata records (25-75%);<br>2: Basic identifier system exists (e.g., internal database IDs) that provides uniqueness within the system;<br>1: No systematic identification system implemented

RDA-F1-01D	Data is identified by a persistent identifier	4 (each experiment can be downloaded from EURISCO incl. a unique experiment_id); 1 (otherwise, because experiment_id cannot be searched for)	2	2	2	System-wide assessment of persistent identifier implementation for C&E datasets	5: All data has PIDs with version history and bidirectional linkage capabilities;<br>4: Majority of data (>75%) has persistent identifiers with global resolution;<br>3: Initial implementation of persistent identifiers for some data (25-75%);<br>2: Basic identifier system exists (e.g., internal database IDs) that provides uniqueness within the system;<br>1: No systematic identification system implemented
RDA-F1-02M	Metadata is identified by a globally unique identifier	1 (dito)	1	1	1	Evaluation of global uniqueness of C&E metadata identifiers across research repositories	5: All metadata has globally unique IDs with cross-system resolution;<br>4: All metadata has globally unique identifiers;<br>3: Most metadata has unique identifiers;<br>2: Some unique identification;<br>1: No globally unique identifiers

RDA-F1-02D	Data is identified by a globally unique identifier	1 (C&E data in EURISCO is not a citable data publication)	1	3	1	Evaluation of global uniqueness of C&E dataset identifiers	5: All data has globally unique IDs with cross-system resolution;<br>4: All data has globally unique identifiers;<br>3: Most data has unique identifiers;<br>2: Some unique identification;<br>1: No globally unique identifiers
RDA-F2-01M	Rich metadata is provided to allow discovery	1	1	1	1	Assessment of experimental design, methodology, and trait definition metadata	5: Complete domain-specific metadata with extended descriptors;<br>4: Full compliance with relevant standards;<br>3: Most required fields completed;<br>2: Basic metadata only;<br>1: Minimal metadata
RDA-F3-01M	Metadata includes the identifier for the data	1	1	1	1	Verification of explicit links between experimental metadata and dataset identifiers	5: Bidirectional linkage with version tracking;<br>4: Clear reference to all data identifiers;<br>3: Most metadata connected to identifiers;<br>2: Basic connection exists;<br>1: No identifier inclusion

RDA-F4-01M	Metadata is offered in such a way that it can be harvested and indexed	2	2	2	2	Assessment of C&E data harvesting mechanisms including trait-specific indexing	5: Multiple harvesting protocols with automated synchronization;<br>4: Standard harvesting mechanisms implemented;<br>3: Basic harvesting capability;<br>2: Limited harvesting support;<br>1: No harvestability
**Accessibility**							
RDA-A1-01M	Metadata contains information to enable the user to get access to the data	1	1	1	1	Evaluation of access information for experimental datasets	5: Complete access instructions for all resource types; 4: Clear access information provided;<br>3: Basic access information;<br>2: Limited access information;<br>1: No access information
RDA-A1-02M	Metadata can be accessed manually (i.e. with human intervention)	3	3	3	3	Assessment of human-readable interfaces for C&E data	5: Advanced user interfaces with domain-specific features;<br>4: Full manual access implementation;<br>3: Basic manual access capability;<br>2: Limited manual access;<br>1: No manual access

RDA-A1-02D	Data can be accessed manually (i.e. with human intervention)	5	5	5	5	Assessment of human interfaces for accessing C&E datasets	5: Advanced interfaces with data type-specific features;<br>4: Complete manual access implementation;<br>3: Basic manual access capability;<br>2: Limited manual access;<br>1: No manual access
RDA-A1-03M	Metadata identifier resolves to a metadata record	1	1	1	1	Testing of identifier resolution for C&E metadata	5: Direct resolution with content negotiation;<br>4: Reliable resolution to complete records;<br>3: Basic resolution functionality;<br>2: Limited resolution capability;<br>1: No resolution implemented
RDA-A1-03D	Data identifier resolves to a digital object	3	3	3	3	Testing of identifier resolution for C&E datasets	5: Direct resolution with content negotiation;<br>4: Reliable resolution to digital objects;<br>3: Basic resolution functionality;<br>2: Limited resolution capability;<br>1: No resolution implemented
RDA-A1-04M	Metadata is accessed through standardised protocol	1	1	1	1	Evaluation of protocols for accessing C&E metadata	5: Multiple standardized protocols with documentation;<br>4: Standard protocol implementation;<br>3: Basic protocol standardization;<br>2: Limited protocol standardization;<br>1: No standardized protocol

RDA-A1-04D	Data is accessible through standardised protocol	1 (just by a web application)	1	1	data retrieval is via HTTPS	Evaluation of protocols for accessing complete C&E datasets	5: Multiple standardized protocols with data-type specific endpoints;<br>4: Standard protocol implementation;<br>3: Basic protocol standardization;<br>2: Limited protocol standardization;<br>1: No standardized protocol
RDA-A1-05D	Data can be accessed automatically (i.e. by a computer program)	1	1	1	1	Assessment of APIs and programmatic access to C&E data	5: Comprehensive API with data-type specific query capabilities;<br>4: Full programmatic access implementation;<br>3: Basic programmatic access;<br>2: Limited automatic access;<br>1: No programmatic access
RDA-A1.1-01M	Metadata is accessible through a free access protocol	2	2	1	2	Verification of free access to C&E metadata	5: Multiple free access protocols with documentation; 4: Free access protocol fully implemented; 3: Basic free access implemented; 2: Limited free access; 1: No free access protocol
RDA-A1.1-01D	Data is accessible through a free access protocol	4 (if we consider the web application a free access protocol)	4	3	4	Verification of free access to C&E datasets	5: Free access with documentation of any limitations;<br>4: Free access protocol fully implemented;<br>3: Basic free access implemented;<br>2: Limited free access;<br>1: No free access protocol

RDA-A1.2-01D	Data is accessible through an access protocol that supports authentication and authorisation	1 (all data freely available by definition)	1	N/A	1	Assessment of authentication for restricted C&E data	5: Granular authentication reflecting legal frameworks; 4: Full authentication implementation where needed; 3: Basic authentication capability; 2: Limited authentication support; 1: No authentication support
RDA-A2-01M	Metadata is guaranteed to remain available after data is no longer available	5 (yes, in the EURISCO backend)	5	1	5	Verification that C&E metadata persists when datasets are archived	5: Complete historical preservation with status indicators;<br>4: Full metadata persistence guarantee;<br>3: Basic metadata preservation;<br>2: Limited persistence guarantee;<br>1: No persistence guarantee
**Interoperability**							
RDA-I1-01M	Metadata uses knowledge representation expressed in standardised format	1	1	1	1	Evaluation of standardized formats (MIAPPE) for C&E metadata	5: Multiple standardized formats with schema definitions;<br>4: Standardized format fully implemented;<br>3: Basic standardized format used;<br>2: Limited standardization;<br>1: No standardized format

RDA-I1-01D	Data uses knowledge representation expressed in standardised format	4	4	1 (are the data in a accepted standardized representation?)	4	Assessment of standardized data formats for phenotypic/genotypic data	5: Multiple standardized formats for different data aspects;<br>4: Standardized format fully implemented;<br>3: Basic standardized format used;<br>2: Limited standardization;<br>1: No standardized format
RDA-I1-02M	Metadata uses machine-understandable knowledge representation	1	1	1	1	Evaluation of machine-readable formats for C&E metadata	5: Formal semantic representation with domain ontologies;<br>4: Machine-understandable format implemented;<br>3: Basic machine readability;<br>2: Limited machine understanding;<br>1: No machine-understandable representation
RDA-I1-02D	Data uses machine-understandable knowledge representation	2	2	2	2	Assessment of machine-readable formats for trait data and methods	5: Formal semantic representation of complex relationships;<br>4: Machine-understandable format implemented;<br>3: Basic machine readability;<br>2: Limited machine understanding;<br>1: No machine-understandable representation

RDA-I2-01M	Metadata uses FAIR-compliant vocabularies	2	2	2	2	Evaluation of controlled vocabularies for traits, methods, scales	5: Multiple FAIR-compliant vocabularies with documentation;<br>4: FAIR-compliant vocabularies used consistently;<br>3: Basic vocabulary standardization;<br>2: Limited vocabulary compliance;<br>1: No FAIR-compliant vocabularies
RDA-I2-01D	Data uses FAIR-compliant vocabularies	2	2	2	2	Assessment of FAIR vocabulary usage in trait definitions and values	5: Comprehensive FAIR vocabularies across all aspects;<br>4: FAIR-compliant vocabularies used consistently;<br>3: Basic vocabulary standardization;<br>2: Limited vocabulary compliance;<br>1: No FAIR-compliant vocabularies
RDA-I3-01M	Metadata includes references to other metadata	1	1	1	1	Verification of references to related metadata (experiments, publications)	5: Comprehensive references to related metadata; 4: Clear references to other metadata; 3: Basic references implemented; 2: Limited references; 1: No references to other metadata
RDA-I3-01D	Data includes references to other data	2	2	3	2	Assessment of references between related datasets or observations	5: Comprehensive references with relationship types;<br>4: Clear references to other data;<br>3: Basic references implemented;<br>2: Limited references;<br>1: No references to other data

RDA-I3-02M	Metadata includes references to other data	1	1	1	1	Verification that C&E metadata references raw datasets	5: Comprehensive references to diverse data types;<br>4: Clear references to related data;<br>3: Basic data references;<br>2: Limited data references;<br>1: No references to data
RDA-I3-02D	Data includes qualified references to other data	2	2	2	2	Assessment of qualified references in C&E data (methodology relations)	5: Comprehensive qualified references with relationship model;<br>4: Qualified references consistently implemented;<br>3: Basic qualified references;<br>2: Limited qualification;<br>1: No qualified references
RDA-I3-03M	Metadata includes qualified references to other metadata	1	1	1	1	Verification of qualified metadata references in C&E documentation	5: Comprehensive qualified references with relationship types;<br>4: Qualified metadata references implemented;<br>3: Basic qualified references;<br>2: Limited qualification;<br>1: No qualified references

RDA-I3-04M	Metadata include qualified references to other data	1	1	1	1	Assessment of qualified references from C&E metadata to diverse data types	5: Comprehensive qualified references with specific semantics;<br>4: Qualified data references implemented;<br>3: Basic qualified references;<br>2: Limited qualification;<br>1: No qualified references
**Reusability**							
RDA-R1-01M	Plurality of accurate and relevant attributes are provided to allow reuse	2	2	2	2	Evaluation of attribute completeness for C&E reuse (analysis, comparison)	5: Comprehensive attributes across all dimensions;<br>4: Multiple relevant attributes provided;<br>3: Basic reuse attributes;<br>2: Limited attribute plurality;<br>1: Insufficient attributes for reuse
RDA-R1.1-01M	Metadata includes information about the licence under which the data can be reused	1	1	1	1	Assessment of licensing information for C&E data	5: Comprehensive licensing for data and material;<br>4: Clear license information provided;<br>3: Basic license information;<br>2: Limited license information;<br>1: No license information

RDA-R1.1-02M	Metadata refers to a standard reuse licence	1	1	1	1	Verification of standard license references for C&E data	5: References to both data licenses and material agreements;<br>4: Standard license references implemented;<br>3: Basic license references;<br>2: Limited standardization;<br>1: No standard license
RDA-R1.1-03M	Metadata refers to a machine-understandable reuse licence	1	1	1	1	Evaluation of machine-readable licenses for C&E data	5: Machine-readable licenses for all aspects;<br>4: Machine-understandable license implemented;<br>3: Basic machine-readable elements;<br>2: Limited machine readability;<br>1: No machine-understandable license
RDA-R1.2-01M	Metadata includes provenance information according to community-specific standards	1 (not in the metadata in the strict sense)	1	1	1	Assessment of experimental methodology and data processing provenance	5: Comprehensive provenance following standards;<br>4: Community-standard provenance implemented;<br>3: Basic provenance information;<br>2: Limited provenance;<br>1: No community-specific provenance

RDA-R1.2-02M	Metadata includes provenance information according to a cross-community language	1	1	1	1	Evaluation of cross-domain provenance for C&E documentation	5: Cross-community provenance with extensions;<br>4: Cross-community provenance implemented;<br>3: Basic cross-community elements;<br>2: Limited cross-community language;<br>1: No cross-community provenance
RDA-R1.3-01M	Metadata complies with a community standard	1 (if we focus on the metadata in the EURISCO backend only)	1	1	1	Verification of MIAPPE compliance for C&E metadata	5: Full compliance with multiple standards;<br>4: Community standard fully implemented;<br>3: Basic standard compliance;<br>2: Limited compliance;<br>1: No community standard compliance
RDA-R1.3-01D	Data complies with a community standard	2 (no widely acknowledged standard existing; EURISCO-own standard)	2	2	2	Assessment of C&E data compliance with experiment standards	5: Compliance with multiple data-type standards;<br>4: Community standard fully implemented;<br>3: Basic standard compliance;<br>2: Limited compliance;<br>1: No community standard compliance

RDA-R1.3-02M	Metadata is expressed in compliance with a machine-understandable community standard	1	1	1	1	Evaluation of machine-readable C&E metadata standard implementation	5: Machine-understandable with validation tools;<br>4: Machine-understandable standard implemented;<br>3: Basic machine-understandable elements;<br>2: Limited machine understanding;<br>1: No machine-understandable standard
RDA-R1.3-02D	Data is expressed in compliance with a machine-understandable community standard	3 (limited)	3	2	3	Assessment of machine-readable C&E data standard compliance	5: Machine-understandable with semantic richness;<br>4: Machine-understandable standard implemented;<br>3: Basic machine-understandable elements;<br>2: Limited machine understanding;<br>1: No machine-understandable standard
